# A Lightweight Multi-Scale Convolutional Network with Gramian Angular Field Encoding for VOC Classification

**DOI:** 10.3390/s26154810

**Published:** 2026-07-29

**Authors:** Yueran Xu, Hanbo Gong, Qing Chen, Mengjiao Shen

**Affiliations:** College of Information, Mechanical and Electrical Engineering, Shanghai Normal University, Shanghai 201418, China; 1000553028@smail.shnu.edu.cn (Y.X.); 1000573894@smail.shnu.edu.cn (H.G.); 1000554353@smail.shnu.edu.cn (Q.C.)

**Keywords:** electronic nose, gas classification, Gramian Angular Summation Field, lightweight neural network, multi-scale depthwise convolution, volatile organic compounds

## Abstract

Accurate classification of volatile organic compounds (VOCs) is important for environmental monitoring and industrial safety via electronic nose (E-nose) systems. However, extracting discriminative features from dynamic one-dimensional sensor responses remains challenging, especially when the recognition model is expected to maintain low computational complexity. This study introduces MSD-GasNet, a lightweight multi-scale depthwise convolutional network combined with Gramian Angular Summation Field (GASF) encoding, for VOC classification using E-nose response signals. The gas-sensing response curves are first transformed into two-dimensional GASF images to preserve temporal correlation information and provide structured inputs for convolutional feature learning. MSD-GasNet further adopts parallel 3 × 3 and 5 × 5 depthwise convolutional branches with feature fusion to capture local response details and broader morphology-related patterns while reducing parameter redundancy. Evaluated on Dataset 1, which contains five representative VOC categories including 1-butanol, acetone, benzaldehyde, butyl acetate, and dimethylbenzene, MSD-GasNet achieves an accuracy of 96.80 ± 0.78%, with 796.6 K parameters and 2.54 ms inference time per sample. Compared with traditional machine learning classifiers, conventional CNN baselines, recent lightweight networks, and a single-scale ablation model, MSD-GasNet shows better classification performance under the current five-class setting. An additional independent validation on Dataset 2 achieves an accuracy of 95.12 ± 1.11% under a chronological train/test split, further supporting the generalization potential of the proposed method. This work provides a GASF-based lightweight multi-scale framework with potential for efficient VOC recognition in portable or resource-limited E-nose applications.

## 1. Introduction

Volatile organic compounds (VOCs) are common gaseous emissions in industrial production and environmental monitoring. VOC detection and discrimination have been studied in many fields, including environmental monitoring, food quality assessment, medical screening, and odor-related management [1,2,3,4]. In many field scenarios, VOCs may be released from solvent use, material processing, coating, storage, cleaning, or leakage. These gases are related to air quality, and they may also reflect process changes, abnormal odors, equipment problems, or early safety risks. For this reason, industrial VOC monitoring needs more than gas detection; it also needs gas-category recognition, so that rapid screening, source identification, and safety warning can be supported. Typical VOC targets in these scenarios may include alcohols, ketones, aldehydes, esters, and aromatic hydrocarbons, which are often related to solvent use, coating and ink emissions, fragrance and fine chemical production, petrochemical processes, and indoor air pollution. These chemically diverse VOC categories may produce different dynamic response patterns in sensor arrays, making them useful targets for evaluating electronic-nose-based gas recognition methods.

Conventional analytical instruments, such as gas chromatography and mass spectrometry, can provide reliable VOC identification, but these instruments usually need expensive equipment, sample preparation, and trained operators. These conditions make them less suitable for continuous, real-time, and portable field monitoring. Electronic nose (E-nose) systems provide another way for on-site VOC recognition. An E-nose system usually combines gas-sensitive sensors, signal acquisition, data processing, and pattern recognition methods, and recent reviews have shown its use in practical sensing and analytical tasks [5,6,7,8]. In an E-nose system, the sensor array records response signals caused by gas exposure, and the recognition model gives the gas category. This working mode makes E-nose systems more suitable for compact and low-cost monitoring tasks.

For practical E-nose systems, gas recognition performance depends not only on sensing materials and sensor arrays, but also on the efficiency and reliability of sensor-signal processing. MOS gas sensor arrays usually generate dynamic response curves during gas exposure, response, and recovery processes, and these signals may be affected by sensor noise, concentration variation, environmental fluctuation, and long-term drift. Therefore, an effective recognition module should be able to make full use of dynamic response information while maintaining low model complexity and fast inference speed for portable or resource-limited E-nose platforms. From this perspective, this study is positioned as an efficient sensing-signal representation and lightweight classification framework for intelligent E-nose systems, rather than as a general image-classification or purely machine-learning-oriented pattern-recognition task.

However, E-nose-based VOC classification still has some problems. Gas sensor responses are usually dynamic one-dimensional curves, not fixed static features. Different VOCs may have similar response trends, and the useful differences may only appear in response amplitude, peak change, or recovery shape. In addition, gas sensor arrays often show cross-sensitivity, so the recognition model needs to extract useful category information from overlapped sensor responses [7]. Handcrafted features may lose these small temporal differences, while large deep learning models may bring too many parameters and high computational cost. These problems are not suitable for portable or resource-limited E-nose platforms; therefore, a useful VOC classification model should keep temporal response information, extract discriminative patterns from small dynamic changes, and maintain a compact structure for efficient inference.

Conventional gas recognition methods usually rely on handcrafted features extracted from sensor response curves, such as maximum response, response/recovery time, slope-related descriptors, integral features, and statistical parameters [9,10,11]. These features are often combined with conventional classifiers, including support vector machines, k-nearest neighbors, logistic regression, random forest, decision trees, and ensemble learning methods. Previous E-nose studies have also shown that machine learning methods can achieve effective VOC discrimination and environmental gas monitoring when the target gases show distinct response patterns [12,13]. However, these methods usually depend on predefined features and specific preprocessing strategies, which may overlook important temporal evolution, peak-region information, recovery-stage behavior, and multi-scale response variations. Therefore, their generalization ability may be limited in complex sensing scenarios, especially when sensor responses are affected by noise, concentration variation, sensor drift, environmental fluctuation, or device-dependent differences.

With the rapid development of machine learning and deep learning, data-driven pattern recognition has been increasingly adopted in smart E-nose systems. Compared with conventional machine learning models, neural networks can automatically learn discriminative representations from data and reduce the dependence on manual feature engineering [14,15,16,17,18]. Convolutional neural networks (CNNs), in particular, have shown strong potential in gas recognition because of their ability to extract local patterns from structured inputs [19]. However, raw gas sensor responses are inherently one-dimensional time-series signals. Directly feeding such sequences into deep models may limit the exploitation of structural information, especially when different VOCs exhibit similar response trends. Moreover, simply increasing the depth or width of CNNs to improve recognition accuracy often introduces a large number of trainable parameters and high computational cost, which is unfavorable for portable or embedded E-nose devices. Thus, an effective VOC classification model should not only extract discriminative response features from gas-sensing signals, but also maintain a simple and efficient architecture with low parameter redundancy and fast inference capability.

To enhance the representation of gas response signals, time-series imaging methods provide a feasible way to transform one-dimensional sensor responses into structured two-dimensional inputs for image-based deep learning models. Gramian Angular Field (GAF) is therefore adopted as a suitable temporal-to-spatial encoding framework, which represents temporal correlations in a Gramian matrix and converts dynamic response patterns into image-like structures. The original GAF framework includes Gramian Angular Summation Field (GASF) and Gramian Angular Difference Field (GADF). In this study, GASF is adopted as the specific encoding strategy because it can map temporal dependencies, local response variations, and overall response morphology into structured visual patterns, allowing CNNs to extract more informative features from sensor responses. Recent studies have demonstrated the feasibility of combining E-nose data, GASF-based image conversion, and CNN models for gas identification and odor intensity classification [20,21]. These studies indicate that image-based representations can improve the utilization of temporal correlation information and provide a more suitable input format for convolutional feature extraction.

Nevertheless, existing CNN-based E-nose recognition methods still face several limitations [22]. First, VOC response images often contain discriminative information at multiple temporal and spatial scales. A single convolutional kernel may be insufficient to capture both short-range local variations and broader response patterns. Second, many CNN models improve feature extraction capability at the expense of model size and inference efficiency, which restricts their application in low-power or resource-constrained E-nose systems. Third, practical VOC classification requires models that can maintain stable performance while avoiding excessive computational burden. Recent studies on sensor-image-based VOC classification, GAF-based gas identification, and lightweight gas recognition have emphasized that high computational complexity, large parameter size, and hardware resource requirements remain major obstacles for practical deployment [23,24,25,26,27,28]. These challenges motivate the development of a lightweight multi-scale CNN that can extract rich discriminative information from GASF images while maintaining computational efficiency.

To address these issues, this study proposes MSD-GasNet, a lightweight multi-scale convolutional neural network that integrates GASF-based response representation with efficient feature extraction for electronic-nose-based VOC classification. This work focuses on developing a sensor-oriented and task-adapted classification framework for dynamic gas-sensing response signals. Raw gas sensor response signals are first transformed into two-dimensional GASF images, and the proposed network is then used to extract discriminative features from different receptive fields [29,30,31,32,33,34,35]. Unlike conventional CNN-based methods that often improve feature representation by increasing network depth or width, the proposed model adopts a lightweight multi-scale design to achieve both compactness and feature diversity [36,37,38]. Specifically, depthwise convolution is used instead of standard convolution to reduce parameter redundancy and computational cost, while parallel convolutional branches are employed to capture local response details and broader morphology-related patterns from GASF images [39,40,41]. Efficient feature fusion is further introduced to integrate these complementary features without introducing excessive computational burden [42,43]. In this way, MSD-GasNet aims to improve VOC classification performance while maintaining the compact structure and efficient inference capability required for practical electronic-nose applications.

The main contributions of this work are summarized as follows:

(1) A GASF-based sensing-signal representation VOC classification framework is constructed, in which one-dimensional gas sensor response signals are transformed into two-dimensional image representations to enhance temporal correlation modeling and structural feature expression of dynamic E-nose responses.
(2) A lightweight multi-scale convolutional neural network is designed to capture discriminative gas response patterns from different receptive fields while reducing model complexity and computational cost. This design is intended to capture both local response details and broader morphology-related patterns without relying on excessive network depth or width.
(3) Comparative experiments are conducted using traditional machine learning classifiers, conventional CNN baselines, recent lightweight networks, and an ablation model to evaluate the proposed method in terms of classification accuracy, parameter scale, and inference efficiency. The results demonstrate that MSD-GasNet achieves an effective trade-off between recognition performance and model complexity, showing its potential for efficient VOC classification in portable and resource-limited electronic-nose systems.

## 2. Data Processing

The gas-sensing data used in this study were derived from the electronic-nose dataset reported by Ni et al. [29], which is referred to as Dataset 1 in this work. In the original work, twelve VOCs were measured using an electronic-nose system equipped with an eight-channel sensor array, and the sensor responses were recorded during repeated gas exposure and recovery processes. Each VOC was tested at concentrations ranging from 10 to 100 ppm, with fifteen repeated experiments for each gas category. Therefore, Dataset 1 provides a systematic benchmark for evaluating electronic-nose-based VOC classification over a wide concentration range. The detailed sensor types and manufacturers used in the sensor array of Dataset 1 are summarized in Table S1 of the Supporting Information.

Based on Dataset 1, this study selected five representative VOCs for classification, including 1-butanol (1BA), acetone (AC), benzaldehyde (BZ), butyl acetate (BAC), and dimethylbenzene (DMB). These five VOCs were selected because they cover different chemical categories and have practical relevance in environmental and industrial monitoring. They include alcohols, ketones, aldehydes, esters, and aromatic hydrocarbons, which may produce different response patterns in a sensor array. In terms of application, 1-butanol, acetone, and butyl acetate are typical solvent-related VOCs; benzaldehyde is related to flavor, fragrance, and fine chemical production; and dimethylbenzene is associated with petrochemical processes, fuel evaporation, and BTEX-type pollution. Therefore, the selection of these five VOCs provides a chemically diverse and application-relevant classification task for examining whether the proposed GASF-based lightweight multi-scale network can effectively learn discriminative dynamic response patterns from different VOC categories. Representative 64 × 64 GASF images of the five VOC categories are provided in Figure S1 of the Supporting Information to further illustrate the category-dependent texture patterns generated by the same image-construction procedure.

It is worth noting that, according to the supplementary information of Ni et al. [29], each VOC gas in Dataset 1 was tested fifteen times in succession before moving on to the next gas. Because the repeated measurements of each VOC in Dataset 1 were conducted successively, possible correlations among repeated observations and time-dependent response variations cannot be completely excluded. To address this limitation, an additional public E-nose dataset reported by Wang et al. [30] was introduced as Dataset 2 for independent validation.

Dataset 2 was collected using an automated unmanned gas-sensing measurement system and originally contains measurements of 12 VOCs. In the gas-sensing experiments, the concentration of each gas was gradually increased from 10 ppm to 100 ppm with a step of 10 ppm, and complete response and recovery curves were recorded. After a complete experiment for one gas was finished, the system was stopped for more than 2 h until the E-nose returned to the idle state before testing the next gas. After all gases were tested in one complete round, the system usually stopped for approximately 3 days before the next experimental round. The experiments were repeated ten times and lasted for about two months. Compared with Dataset 1, this experimental protocol provides better temporal and experimental independence because repeated measurements were collected across different rounds and time periods. In the independent validation experiment, the first five complete experimental rounds of Dataset 2 were used as the training set, while the last five complete experimental rounds were used as the testing set. This chronological splitting strategy avoids randomly mixing samples from the same experimental round into both the training and testing sets.

To keep the additional validation task comparable with Dataset 1, five representative VOCs were selected from Dataset 2, namely acetone, benzaldehyde, butyl acetate, dimethylbenzene, and methanol. Among them, four VOCs overlap with the categories used in Dataset 1, while methanol was selected as the representative alcohol compound in Dataset 2.

**Figure 1:**
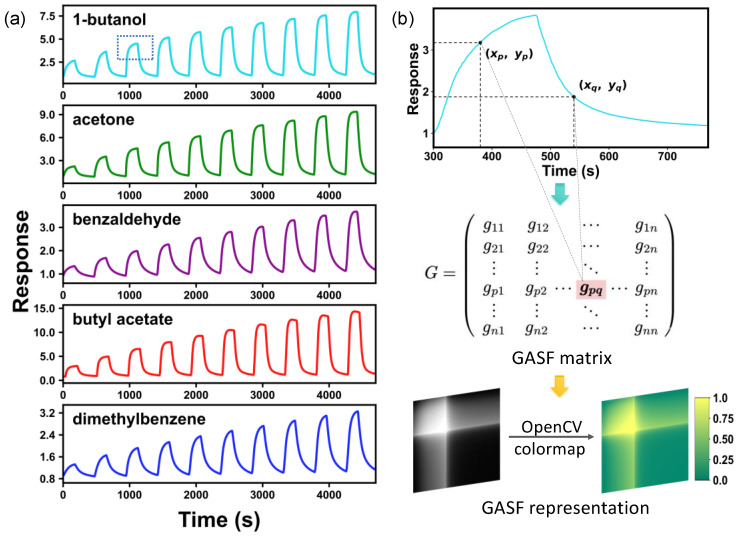
GASF-based image construction process for VOC classification. (**a**) Raw response curves of five VOCs. (**b**) Schematic illustration of fixed response segmentation and its transformation into a GASF matrix and two-dimensional image.

As shown in Figure 1a, the five selected VOCs exhibit distinguishable response patterns in terms of response amplitude, peak evolution, curve morphology, and recovery behavior, which provide discriminative information for classification. However, raw response curves are one-dimensional time-series signals and are not directly optimized for CNN-based feature extraction. Therefore, GASF encoding was adopted to transform the selected response segments into structured two-dimensional representations. Figure 1b illustrates the GASF-based image construction process used in this study. Before GASF transformation, response segments were extracted using a fixed segmentation rule. The starting points were set to sample point 600 for benzaldehyde (BZ), sample point 1100 for butyl acetate (BAC), and sample point 0 for 1-butanol (1BA), acetone (AC), and dimethylbenzene (DMB). From each starting point, up to 9400 consecutive sampling points were retained and divided into non-overlapping segments of 940 points, while incomplete tail segments were discarded. The same rule was applied to all training and testing samples within each VOC category, and no sample-specific manual selection was performed. Each response segment was then converted into a two-dimensional GASF representation for subsequent CNN-based feature learning. Through this process, the original one-dimensional gas response sequence is transformed into a structured image input for subsequent convolutional feature extraction.

For each gas sample, the response sequence was extracted from the raw sensor curve using the fixed segmentation rule described above. The extracted response sequence can be denoted as $x=\left[x_{1},x_{2},x_{3},\ldots \ldots ,x_{N}\right]$ where $x_{i}$ represents the response value at the i-th sampling instant, and N denotes the sequence length. In this study, the response segment was generated by a consistent rule rather than by manually selecting the rising stage, peak region, or recovery stage. This strategy avoids sample-specific manual cropping and provides fixed-length input sequences for subsequent GASF image construction.

Before Gramian Angular Field encoding, each response sequence was normalized into the interval $\left[0,\;1\right]$. This normalization is necessary for the subsequent angular mapping and also reduces the influence of scale differences among different response curves. For a given sequence x, the normalized $\tilde{x_{i}}$ is calculated as

(1) $$x_{i}^{\prime }=\frac{x_{i}-\min\left(X\right)}{\max\left(X\right)-\min\left(X\right)}$$

where min(x) and max(x) represent the minimum and maximum values of the selected response sequence, respectively. It should be noted that this normalization is used for image encoding rather than for replacing the physical meaning of the original response curve. The relative variation trend of the sensing signal is preserved after normalization.

To convert the normalized one-dimensional sequence into a two-dimensional representation, Gramian Angular Summation Field (GASF) encoding was adopted in this study. GASF is a specific form of Gramian Angular Field that maps a time series into an angular space and constructs a matrix by calculating the angular relationship between different sampling points. After normalization, each response value is first transformed into an angular representation:

(2) $$\Phi_{i}=\arccos\left(\tilde{x_{i}}\right)$$

$\Phi_{i}$ is the angular value corresponding to the i-th sampling instant. For any two sampling points i and j, the corresponding GASF matrix element is obtained by

(3) $$G_{i,j}^{GASF}=\cos\left(\Phi_{i}+\Phi_{j}\right)$$

This expression can also be written as

(4) $$G_{i,j}^{\mathrm{GASF}}=\tilde{x_{i}}\tilde{x_{j}}-\sqrt{1-\tilde{x_{i}^{2}}\sqrt{1-\tilde{x_{j}^{2}}}}$$

Through this operation, a one-dimensional sequence of length N is transformed into an N×N matrix:

(5) $$G=\left[\begin{array}{cccc} g_{11} & g_{12} & \ldots & g_{1N}\\ g_{21} & g_{22} & \ldots & g_{2N}\\ \vdots & \vdots & \ddots & \vdots \\ g_{N1} & g_{N2} & \ldots & g_{NN} \end{array}\right]$$

Each element $g_{ij}$ encodes the relationship between the i-th and j-th sampling instants. Therefore, the GASF matrix is not merely a visual transformation of the original curve, but a structured representation that preserves temporal dependency and global response information. Compared with the original one-dimensional signal, this representation makes the correlation patterns among sampling points more accessible to convolutional operations, thereby facilitating the extraction of response evolution features for VOC classification.

After GASF encoding, the matrix was further visualized as a two-dimensional image. The intensity distribution of the image reflects the pairwise relationships among different sampling instants, while the texture structure corresponds to the dynamic evolution of the gas response curve. In this study, the generated GASF image was resized to a fixed resolution of 64 × 64 and converted into RGB format to match the input requirement of the proposed convolutional neural network. The final input image can be represented as $I\in R^{H\times W\times 3}$, where H and W denote the image height and width, respectively. In this work, H=W=64. Each image inherits the class label of the corresponding VOC category and is used for training and testing the classification model.

Through the above processing procedure, the original VOC response signal is transformed from a one-dimensional temporal curve into a structured two-dimensional GASF image. This representation preserves the temporal characteristics of the sensing response and provides an image-based input for MSD-GasNet, a lightweight multi-scale depthwise convolutional network designed for VOC classification.

## 3. Methodology

### 3.1. Model Structure

**Figure 2:**
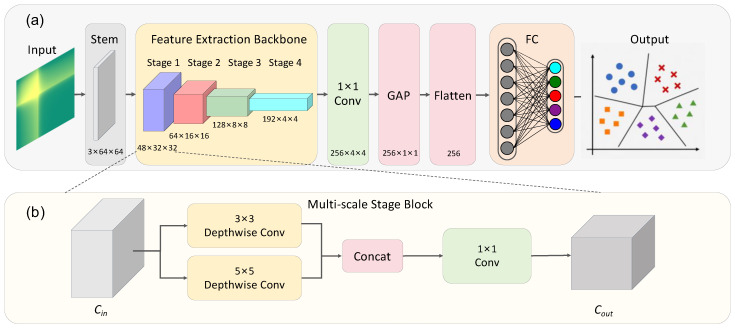
Architecture of MSD-GasNet. (**a**) Overall framework for GASF-based VOC image classification. (**b**) Structure of the multi-scale stage block, including parallel 3 × 3 and 5 × 5 depthwise convolution branches and feature fusion.

The proposed network is designed as a lightweight multi-scale classification model for GASF-based VOC images. As shown in Figure 2, the network adopts a compact hierarchical architecture composed of an initial ConvBNAct layer, four lightweight multi-scale stages, a 1 × 1 convolutional fusion layer, global average pooling, and a final classification head. The hierarchical design enables the model to progressively extract features from low-level texture patterns to high-level response morphology, while the compact structure is achieved by using depthwise convolution in the multi-scale branches and global average pooling before classification. Instead of relying on a heavy backbone with a large number of standard convolutions, the network is organized into staged multi-scale modules, in which parallel depthwise convolution branches are used to describe local and broader response patterns in GASF images. Detailed architectural parameters of MSD-GasNet are provided in Figure S2 of the Supporting Information.

The key design of the proposed model lies in the multi-scale depthwise convolution module. Gas response images generated by GASF encoding contain both local texture variations and broader structural patterns. Local regions may reflect subtle changes in response amplitude or short-term temporal fluctuations, while larger spatial regions usually correspond to the global evolution of the response curve. Therefore, relying on a single convolutional receptive field may be insufficient for capturing discriminative VOC patterns. To address this issue, two parallel depthwise convolutional branches with different kernel sizes are introduced. The 3 × 3 branch focuses on fine-grained local information, while the 5 × 5 branch captures broader response morphology. By combining these two branches, the model can extract complementary features from different receptive fields without introducing a large number of standard convolutional parameters.

To reduce model complexity, depthwise convolution is used in the multi-scale branches. Compared with standard convolution, depthwise convolution performs spatial filtering independently for each channel, which significantly decreases the number of parameters and computational operations. After multi-scale feature extraction, the network uses channel fusion and subsequent convolutional refinement to enhance feature interaction. The extracted feature maps are then aggregated by global average pooling rather than being directly flattened into a large fully connected layer. This operation compresses the spatial information into a compact feature vector, reduces parameter redundancy, and improves the robustness of the classifier. Finally, the classification head maps the learned representation to the target VOC categories and outputs the predicted class probabilities. Therefore, the proposed architecture is designed to extract multi-scale discriminative features from GASF-based VOC images while maintaining a compact parameter scale for efficient classification.

### 3.2. Implementation Details

MSD-GasNet was implemented in PyTorch and trained on a local workstation equipped with an 11th Gen Intel(R) Core(TM) i7-1165G7 CPU. The software environment was Python 3.11.3 with PyTorch 2.11.0+cpu. The source code is publicly available at https://github.com/ziran1234/MSD-GasNet (accessed on 26 July 2026).

A predefined and class-balanced partition protocol was used to ensure reproducible model evaluation. For each VOC category, 12 response files were used for training, and 3 response files were used for testing, corresponding to an 8:2 split ratio. The same strategy was consistently applied to all five classes, ensuring that the training and testing subsets preserved the original class distribution. Mini-batch shuffling was enabled during training to reduce order-dependent optimization bias.

The classification model was trained in a supervised manner using cross-entropy loss. Adam was adopted as the optimizer, and a step-based learning rate decay strategy was applied. The learning rate scheduler updated the learning rate every fixed number of epochs by multiplying it with a decay factor. The hyperparameter settings used for classification training on Dataset 1 are summarized in Table 1. The hyperparameter configurations of all deep learning models used in the independent validation experiment on Dataset 2 are summarized in Table S2 of the Supporting Information.

For the five-class VOC classification task, cross-entropy loss was used to measure the discrepancy between the predicted class probabilities and the ground-truth labels. For a mini-batch containing N samples and C VOC categories, the loss is defined as:

(6) $$L=-\frac{1}{N}\sum_{i=1}^{N}\sum_{c=1}^{C}y_{i,c}\log\left(\hat{y_{i,c}}\right)$$

where $y_{i,c}$ is the one-hot encoded ground-truth label of sample i for class c and ${\hat{y}}_{i,c}$ denotes the predicted probability after softmax normalization. In this study, C=5, corresponding to the five selected VOC categories.

## 4. Results and Discussion

### 4.1. Training Process and Convergence Analysis

**Figure 3:**
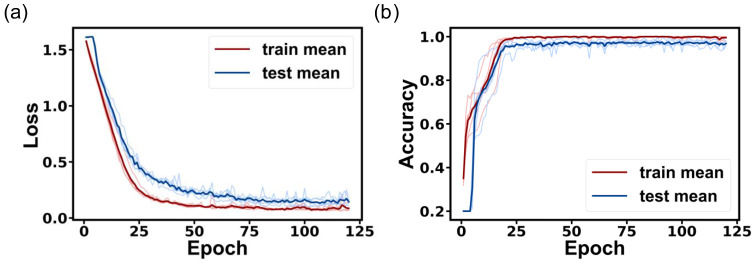
Training process of the proposed model. (**a**) Loss curves of the training and testing sets. (**b**) Accuracy curves of the training and testing sets of Dataset 1.

The training process of the proposed model was first analyzed to check whether the MSD-GasNet model could be optimized in a stable way. Figure 3 shows the loss and accuracy curves obtained from 5-fold cross-validation on Dataset 1. In this figure, the light-colored curves represent the results of each individual fold, while the dark-colored curves represent the mean curves of the five folds on the training and testing sets of Dataset 1. This setting makes it possible to observe both the average training trend and the fluctuation among different folds.

As shown in Figure 3a, the training loss and testing loss both decrease quickly at the early stage of training. The loss drops sharply in the first several epochs, which means that the model can quickly learn useful information from the GASF-based VOC images. After about 25 epochs, the two loss curves become much smoother and then change only slightly. This trend indicates that the network parameters are gradually adjusted to a stable state, and the training process begins to converge. The testing loss is slightly higher than the training loss, but the two curves follow a similar downward trend, and no obvious divergence is observed. This result suggests that the model not only fits the training data, but also keeps a relatively stable performance on the testing data. The small fluctuations in the light-colored curves are normal across different folds, while the mean curves remain smooth, showing that the selected optimizer, learning rate, and learning-rate decay strategy are generally suitable for this task.

The accuracy curves in Figure 3b show a similar pattern. At the beginning of training, the training accuracy increases rapidly, and the testing accuracy also rises quickly after the first few epochs. After the early optimization stage, both curves remain at a high level and show only small changes. The training accuracy is slightly higher than the testing accuracy, but the gap between them is not large, which suggests that severe overfitting is not observed in the current training process. In addition, the accuracy curves from the five folds are close to each other after convergence, indicating that the model can obtain relatively consistent results under different data splits. The training curves of the additional baseline models and the ablation variant on Dataset 1 are provided in Figures S3–S10 of the Supporting Information, including ResNet50, VGG19, MobileNetV3-large, AlexNet, MSD-GasNet 1, GhostNetV3-1.0, StarNet-S2, and FastViT-T8. These curves provide additional references for comparing the convergence behavior of different models under the same GASF-based VOC classification setting. Based on the loss and accuracy curves, MSD-GasNet reaches a stable optimization state before the end of training, which provides a reasonable basis for the following performance comparison.

### 4.2. Comparative Classification Performance Analysis

To evaluate the classification capability of MSD-GasNet, traditional machine learning classifiers, conventional CNN baselines, recent lightweight networks, and an ablation model were selected for comparison. The traditional machine learning classifiers included Logistic Regression (LR), Random Forest (RF), Quadratic Discriminant Analysis (QDA), and k-Nearest Neighbors (KNN). These methods were included to provide conventional feature-based references for the five-class VOC classification task. Four representative CNN models were selected as baselines, including ResNet50, VGG19, MobileNetV3-large, and AlexNet. These models are publicly available and widely used in image classification tasks, providing reproducible references for comparison. They also represent different architectural paradigms: AlexNet represents a classical CNN structure, VGG19 represents deep convolutional stacking with standard convolutions, ResNet50 represents residual learning, and MobileNetV3-large represents lightweight mobile-oriented CNN design.

In addition, GhostNetV3-1.0, StarNet-S2, and FastViT-T8 were further introduced as recent lightweight network baselines to strengthen the comparison with efficient architectures. Since the proposed method converts gas response signals into GASF images, comparing MSD-GasNet with these image classification backbones enables a structurally diverse evaluation rather than a comparison with a single type of network. In addition, to evaluate the contribution of the multi-scale branch structure, an ablation model named MSD-GasNet 1 was constructed. This model retains the basic framework of the main network but replaces the original multi-scale branches with a single-scale depthwise convolutional structure. The classification results are summarized in Table 2, where the mean accuracy and standard deviation were used to assess recognition performance and stability.

As shown in Table 2, MSD-GasNet gives the highest mean accuracy among the compared models, reaching 96.80 ± 0.78%. Among the traditional machine learning classifiers, RF achieves the best performance with an accuracy of 90.67 ± 1.84%, followed by KNN, LR, and QDA. These results indicate that conventional classifiers can obtain reasonable recognition results for the selected five VOCs, but their performance is still lower than that of MSD-GasNet. Compared with the four CNN baseline models, MSD-GasNet increases the mean accuracy by 8.00 percentage points over ResNet50, 9.47 percentage points over VGG19, 15.73 percentage points over MobileNetV3-large, and 5.60 percentage points over AlexNet. MSD-GasNet also outperforms the recent lightweight networks, including GhostNetV3-1.0, StarNet-S2, and FastViT-T8. Moreover, it improves the accuracy by 8.53 percentage points compared with MSD-GasNet 1. This result shows that the multi-scale branch design helps improve the classification result. Since MSD-GasNet 1 only uses a single-scale depthwise convolution structure, the difference between the two models indicates that using multi-scale branches can provide more useful information for learning GASF-based VOC images. These results indicate that the proposed model provides stronger classification performance than the selected traditional machine learning methods, CNN baseline models, recent lightweight networks, and the single-scale ablation model under the current Dataset 1 setting.

In addition to accuracy, MSD-GasNet shows more stable results in repeated experiments. Its standard deviation is 0.78%, which is lower than those of ResNet50, VGG19, MobileNetV3-large, AlexNet, GhostNetV3-1.0, and StarNet-S2, and is close to that of FastViT-T8 and MSD-GasNet 1. This smaller variation suggests that the model is less affected by data partitioning and random training differences, rather than benefiting from a single favorable split or training run. This improvement is mainly due to the parallel depthwise convolutional branches with different receptive fields, which help the model capture both local details and broader response patterns from GASF-based VOC images.

**Figure 4:**
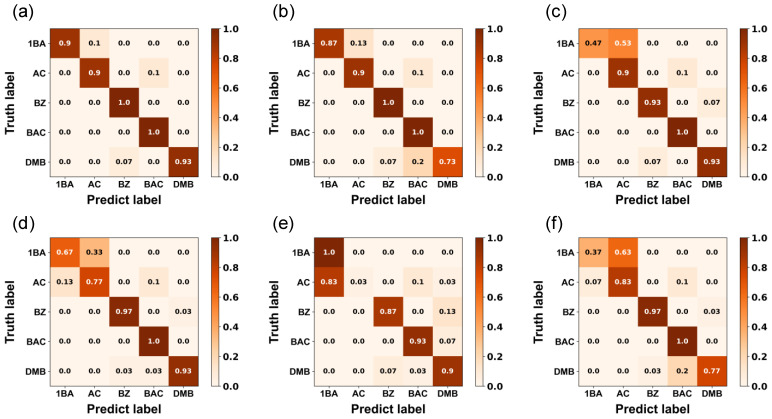
Confusion matrices of different classification models: (**a**) MSD-GasNet, (**b**) MSD-GasNet 1, (**c**) ResNet50, (**d**) VGG19, (**e**) MobilenetV3-large, and (**f**) AlexNet.

At the category level, the confusion matrices of different models on Dataset 1 are shown in Figure 4. Compared with the baseline models, MSD-GasNet shows clearer diagonal dominance for most VOC categories, meaning that more samples are assigned to their correct classes. This result is consistent with the accuracy comparison in Table 2, and it also supports the role of the multi-scale depthwise convolutional design in extracting useful features from GASF-based VOC images. Some misclassifications still occur between categories with similar response patterns, but MSD-GasNet generally keeps better category separation among the five VOCs. This further shows that the proposed model has a stronger classification ability in the current VOC recognition task. The confusion matrices of traditional machine learning classifiers and additional deep learning models on Dataset 1 are provided in Figures S11 and S12 of the Supporting Information.

**Figure 5:**
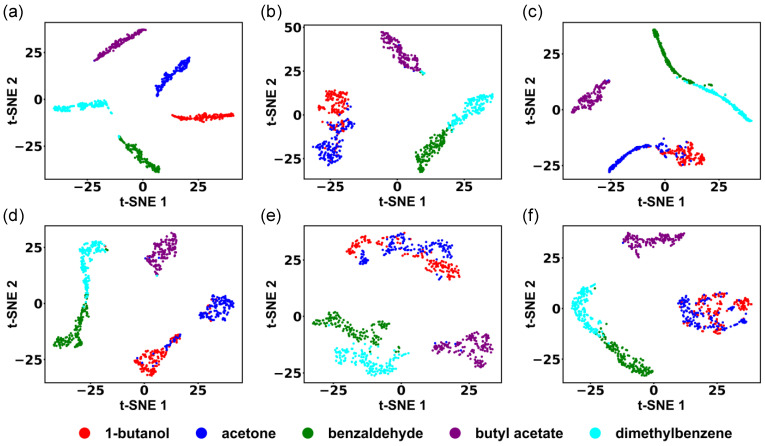
t-SNE visualization of learned feature distributions extracted by different classification models on Dataset 1: (**a**) MSD-GasNet, (**b**) MSD-GasNet 1, (**c**) ResNet50, (**d**) VGG19, (**e**) MobilenetV3-large, and (**f**) AlexNet.

The learned feature distributions on Dataset 1 were further visualized by t-SNE, as shown in Figure 5. For each model, the feature vectors before the final classification layer were used as the input, so that the separability of the learned representations could be compared more directly. In several baseline models and the ablation model, some clusters are relatively scattered, and partial overlap can still be observed between categories with similar response patterns. In contrast, MSD-GasNet shows more compact intra-class clusters and clearer inter-class separation for most VOC categories. This pattern is consistent with the accuracy results and confusion matrix analysis, suggesting that the multi-scale receptive-field design helps the network learn more useful class-related features from GASF-based VOC images. The t-SNE visualizations of additional deep learning models on Dataset 1 are provided in Figure S13 of the Supporting Information.

To further evaluate the generalization ability of the proposed model under a more independent experimental setting, Dataset 2 was used as an additional validation dataset. The detailed classification results are provided in Table S3 of the Supporting Information. MSD-GasNet achieved the highest accuracy of 95.12 ± 1.11% among the compared traditional machine learning methods, CNN baselines, recent lightweight networks, and the ablation model, further supporting its generalization potential under the chronological train/test split. The corresponding training and testing curves of MSD-GasNet, the ablation model, and the compared deep learning models are provided in Figures S14–S22. These curves further illustrate the convergence behavior of the different models under the chronological train/test split. The confusion matrices of traditional machine learning classifiers and deep learning models are provided in Figures S23 and S24, while the corresponding t-SNE visualization results are provided in Figure S25 of the Supporting Information.

### 4.3. Model Complexity, Computational Efficiency, and Encoding Method Comparison

In addition to classification accuracy, model complexity is a critical factor for electronic nose applications. Since VOC recognition systems are often expected to operate on portable or resource-constrained platforms, the classification model should be both accurate and computationally efficient. Therefore, the number of parameters, training time, and single-sample inference time were compared among different models, as summarized in Table 3.

As shown in Table 3, MSD-GasNet contains only 796.645 K parameters, which is substantially smaller than the baseline networks. Its parameter size is approximately 3.39% of ResNet50, 0.57% of VGG19, 18.93% of MobileNetV3-large, and 1.40% of AlexNet, 11.62% of GhostNetV3-1.0, 23.25% of StarNet-S2, and 24.43% of FastViT-T8. This confirms the lightweight nature of the proposed architecture.

Under the same experimental setting, the proposed model also achieves the shortest training time and inference time among the compared models. Specifically, the training time of MSD-GasNet is 94.001 s, which is lower than those of all compared deep learning models on Dataset 1. More importantly, its single-sample inference time is only 2.537 ms. These results indicate that MSD-GasNet provides a favorable balance between classification performance and computational efficiency, which is important for portable or resource-constrained electronic-nose applications. As shown in Table S4 of the Supporting Information, MSD-GasNet also maintained favorable computational efficiency on Dataset 2.

The lightweight advantage mainly comes from the use of depthwise convolution and compact feature aggregation. Depthwise convolution reduces redundant spatial convolution operations by filtering each channel independently, while the subsequent pointwise convolution performs efficient channel fusion. In addition, global average pooling replaces large fully connected transformations after feature extraction, further reducing parameter redundancy. These designs enable the model to maintain strong classification ability while significantly lowering computational cost.

In addition to model-level efficiency, the influence of the time-series-to-image encoding strategy was also examined. Additional experiments were conducted by comparing different encoding methods, including CWT, MTF, STFT, and GASF. The quantitative comparison is summarized in Table S5 of the Supporting Information, and representative images and training curves are provided in Figures S26–S29. Among the compared encoding methods, GASF achieved the highest and most stable classification accuracy, reaching 96.80 ± 0.78% on Dataset 1. Although STFT showed slightly shorter training and inference times, GASF provided better classification performance with comparable computational cost. Therefore, GASF was selected as the main encoding method in this study.

Finally, to examine the scalability of the proposed framework beyond the selected five representative VOCs, an additional scalability experiment was conducted using all 12 VOC categories in Dataset 1. In this more challenging setting, the number of target classes increased from 5 to 12, and some VOCs may have more similar response characteristics, which makes the classification boundaries more complex. MSD-GasNet achieved a test accuracy of 87.01 ± 2.40%, with an average training time of 195.061 s and a single-sample inference time of 2.494 ms. Although the accuracy decreased compared with the five-class setting, the model still maintained reasonable classification performance under a substantially expanded category set. This result indicates that the proposed GASF-based lightweight multi-scale framework is not limited to the selected five VOCs and has preliminary scalability to a larger VOC classification task. The corresponding training curves, confusion matrix, and t-SNE visualization are provided in Figures S30–S32.

## 5. Conclusions

This study developed MSD-GasNet, a sensor-oriented lightweight multi-scale convolutional network combined with GASF encoding for electronic-nose-based VOC classification. The proposed framework transforms one-dimensional gas sensor responses into two-dimensional GASF images, enabling dynamic response information to be represented in a structured form for convolutional feature learning. By using multi-scale depthwise convolution, MSD-GasNet can extract both local response details and broader morphology-related patterns with low computational cost. Therefore, this work provides a task-adapted sensing-signal representation and lightweight classification framework for dynamic E-nose response analysis.

The experimental results demonstrate that MSD-GasNet achieves an effective balance among classification accuracy, model compactness, and inference efficiency. On Dataset 1, it achieved an accuracy of 96.80 ± 0.78% with 796.6 K parameters and a single-sample inference time of 2.54 ms, outperforming traditional machine learning classifiers, conventional CNN baselines, recent lightweight networks, and the single-scale ablation model. Additional validation on Dataset 2 achieved 95.12 ± 1.11% under a chronological train/test split, supporting the generalization potential of the proposed method under a more independent validation setting. In addition, the encoding comparison further supports the effectiveness of GASF as the time-series-to-image representation used in this framework. Future work will focus on self-collected and randomized experimental datasets, structurally similar VOCs, mixed gases, broader concentration ranges, long-term drift correction, cross-device validation, and real-time deployment on resource-limited electronic-nose platforms.

## Figures and Tables

**Table 1:** Hyperparameter configurations for the training process on Dataset 1.

Hyperparameter	Value
epoch	120
batch size	48
initial learning rate	0.00005
optimizer	Adam
betas	(0.9, 0.999)
weight_decay	0.1
step_size	20
image size	64 × 64

**Table 2:** Results of classification task on Dataset 1.

Model	Accuracy Mean (%)	Accuracy Std. (%)
LR	85.73	1.33
RF	90.67	1.84
QDA	84.13	2.78
KNN	88.67	1.66
ResNet50	88.80	2.25
VGG19	87.33	5.13
MobilenetV3-large	81.07	2.82
AlexNet	91.20	0.98
GhostNetV3-1.0	89.93	2.06
StarNet-S2	86.19	3.11
FastViT-T8	90.08	0.77
MSD-GasNet 1	88.27	0.68
MSD-GasNet	96.80	0.78

**Table 3:** Computational efficiency comparison of different classification models on Dataset 1.

Model	Params.	Training Time (s)	Single Inference Time (ms)
ResNet50	23.5183 M	140.332384	6.440
VGG19	139.5907 M	285.557302	4.833
MobilenetV3-large	4.2084 M	105.097674	7.264
AlexNet	57.0243 M	103.344144	3.173
GhostNetV3-1.0	6.8544 M	274.328325	3.932
StarNet-S2	3.4263 M	103.639428	4.104
FastViT-T8	3.2611 M	119.967555	4.478
MSD-GasNet	796.6450 K	94.001315	2.537

## Data Availability

The original contributions presented in this study are included in the article/Supplementary Materials. Further inquiries can be directed to the corresponding author.

## Supplementary Materials

The following supporting information can be downloaded at: https://www.mdpi.com/article/10.3390/s26154810/s1, Table S1: The sensors used in the sensor array in Dataset 1; Table S2: Hyperparameter configurations of different deep learning models in the independent validation experiment on Dataset 2; Table S3: Classification performance comparison of traditional machine learning methods, CNN baselines, lightweight networks, the ablation model, and MSD-GasNet on Dataset 2; Table S4: Computational efficiency comparison of different deep learning models on Dataset 2; Table S5: Performance comparison of different time-series-to-image encoding methods using the proposed MSD-GasNet on Dataset 1; Figure S1: Representative 64 × 64 GASF images generated from five VOC categories; Figure S2: Detailed architecture of MSD-GasNet; Figures S3–S10: Training processes of baseline models and the ablation model on Dataset 1, including ResNet50, VGG19, MobileNetV3-large, AlexNet, MSD-GasNet 1, GhostNetV3-1.0, StarNet-S2, and FastViT-T8; Figures S11 and S12: Confusion matrices of traditional machine learning classifiers and different deep learning models on Dataset 1; Figure S13: t-SNE visualization of learned feature distributions extracted by different deep learning models on Dataset 1; Figures S14–S22: Training and testing processes of different deep learning models on Dataset 2, including MSD-GasNet, MSD-GasNet 1, ResNet50, VGG19, MobileNetV3-large, AlexNet, GhostNetV3-1.0, StarNet-S2, and FastViT-T8; Figures S23 and S24: Confusion matrices of traditional machine learning classifiers and different deep learning models on Dataset 2; Figure S25: t-SNE visualization of learned feature distributions extracted by different deep learning models on Dataset 2; Figure S26: Representative images generated by different time-series-to-image encoding methods; Figures S27–S29: Training processes of MSD-GasNet using CWT, MTF, and STFT images on Dataset 1; Figures S30–S32: Training process, confusion matrix, and t-SNE visualization of MSD-GasNet for the 12-class VOC classification task on Dataset 1.
