# Supplementary material for: A Lightweight Multi-Scale Convolutional Network with Gramian Angular Field Encoding for VOC Classification
Source: Sensors (Basel). 2026 Jul 29;26(15):4810. doi: 10.3390/s26154810 (PMC13469688; doi:10.3390/s26154810)
Supplement: Supplementary file 1 [file sensors-26-04810-s001.zip › sensors-4391093-supplementary.pdf]

Supporting Information

A Lightweight Multi-Scale Convolutional Network with Gramian Angular Field Encoding for VOC Classification

Yueran Xu , Hanbo Gong , Qing Chen , Mengjiao Shen *

College of Information, Mechanical and Electrical Engineering, Shanghai Normal University, Shanghai 201418, China; 1000553028@smail.shnu.edu.cn (Y.X.); 1000573894@smail.shnu.edu.cn (H.G.); 1000554353@smail.shnu.edu.cn (Q.C.)

* Correspondence: mengjiaos@shnu.edu.cn

Supporting Information S1 – Dataset Description and GASF-Based Image Representation

This section provides additional implementation details for dataset organization, label assignment, and GASF image generation. Five VOC categories were used in the classification task, including 1-butanol, acetone, benzaldehyde, butyl acetate, and dimethylbenzene. The corresponding numerical labels were assigned as 0, 1, 2, 3, and 4, respectively. The same label mapping was used for all training and testing procedures to ensure consistency across different models.

The original response data were organized under gas-specific folders and further divided into training and testing subsets. During image generation, only the selected five VOC categories were processed. For each CSV response file, the response sequence was read from the average response column. To remove non-informative initial regions and keep the main response process, gas-specific starting rows were used before segment extraction. The starting rows were set to 0 for 1-butanol, acetone, and dimethylbenzene, 600 for benzaldehyde, and 1100 for butyl acetate. From the starting row, a maximum response range of 9400 sampling points was considered. If the total sequence length was shorter than 9600 points, the starting row was reset to 0.

The selected response range was divided into fixed-length segments for image construction. Each segment was assigned to a concentration-related folder according to the generated ppm index, increasing from 10 ppm to 100 ppm. For each segment, a GASF matrix was calculated and then linearly normalized to the 0-255 intensity range. The normalized matrix was converted into an unsigned 8-bit image and visualized using a pseudo-color mapping. The generated images were resized to 64×64 pixels and used as CNN inputs. During model loading, each image was further converted into a tensor and normalized before being fed into the classification model.

Representative GASF images of the five VOC categories are shown in Figure S1. These images were generated using the same segment extraction, GASF encoding, intensity normalization, pseudo-color mapping, and resizing procedure. The visual differences among categories indicate that the temporal response patterns of different VOCs can be transformed into distinguishable image textures for convolutional feature learning. The sensor types, manufacturers, and channel labels used in the eight-channel sensor array of Dataset 1 are summarized in Table S1.

Table S1. The sensors used in the sensor array in Dataset 1.

Sensor Name	Type	Company	Label
GM-502B	MOS-MEMS	Winsen Electronics Technology Co., Ltd.	Sen. 1
GM-202B	MOS-MEMS	Winsen Electronics Technology Co., Ltd.	Sen. 2
SMD-1005	MOS-MEMS	Suzhou Huiwen Nano S&T Co., Ltd.	Sen. 3
SMD-1013B	MOS-MEMS	Suzhou Huiwen Nano S&T Co., Ltd.	Sen. 4
MQ-138	MOS-Ceramic Tube	Winsen Electronics Technology Co., Ltd.	Sen. 5
MQ-2	MOS-Ceramic Tube	Winsen Electronics Technology Co., Ltd.	Sen. 6
TGS-2609	MOS-Ceramic Tube	Figaro Engineering Inc.	Sen. 7
TGS-2620	MOS-Ceramic Tube	Figaro Engineering Inc.	Sen. 8

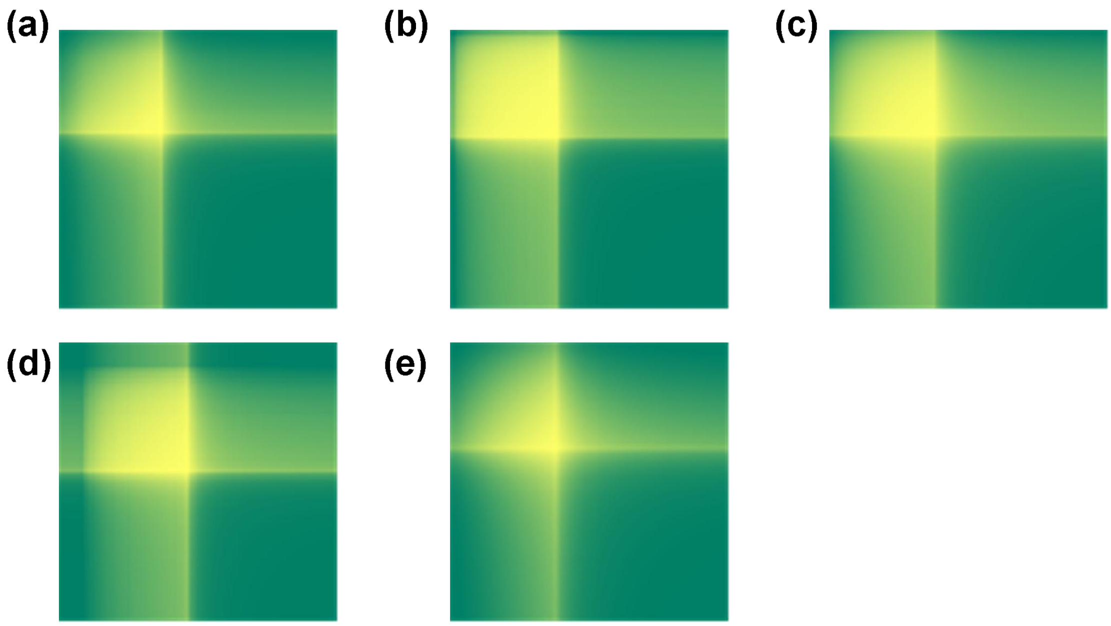

Figure S1. Representative 64×64 GASF images generated from five VOC categories: (a) 1-butanol, (b) acetone, (c) benzaldehyde, (d) butyl acetate, and (e) dimethylbenzene.

Supporting Information S2 - Layer-wise Architecture and Parameter Configuration of MSD-GasNet

MSD-GasNet is designed as a lightweight multi-scale depthwise convolutional network for GASF-based VOC classification. As shown in Figure S2(a), the network takes a normalized three-channel GASF image with a size of 3×64×64 as input. The feature extraction backbone starts with a ConvBNAct stem layer, which maps the input image from 3 channels to 32 channels while maintaining the spatial resolution. Four subsequent lightweight multi-scale depthwise convolutional stages are used to progressively extract hierarchical response-image features. The output channels of Stage 1 to Stage 4 are 48, 64, 128, and 192, respectively, and the stride of each stage is set to 2. Accordingly, the feature-map size is gradually changed from 32×64×64 to 48×32×32, 64×16×16, 128×8×8, and 192×4×4.

After the four feature extraction stages, a 1 × 1 ConvBNAct layer maps the feature channels from 192 to 256, producing a feature map of 256×4×4. Adaptive global average pooling then compresses this feature map to 256×1×1, followed by flattening into a 256-dimensional feature vector. The classification head consists of a dropout layer, a fully connected layer from 256 to 128 dimensions, a SiLU activation function, another dropout layer, and a final fully connected layer from 128 dimensions to five VOC classes.

The internal structure of the lightweight multi-scale depthwise convolutional stage is shown in Figure S2(b). Each stage contains a multi-scale depthwise convolution module followed by a 3 × 3 ConvBNAct refinement layer. Inside the multi-scale depthwise convolution module, a 1×1 ConvBNAct operation is first used to project the input channels to the target channel dimension. The projected feature map is then processed by two parallel depthwise convolution branches with 3×3 and 5×5 kernels. The 3×3 branch focuses on local texture variations in the GASF image, whereas the 5×5 branch captures broader response-pattern structures. The outputs of the two branches are concatenated along the channel dimension and fused by a 1×1

ConvBNAct operation. A subsequent 3×3 ConvBNAct layer further refines the fused feature map.

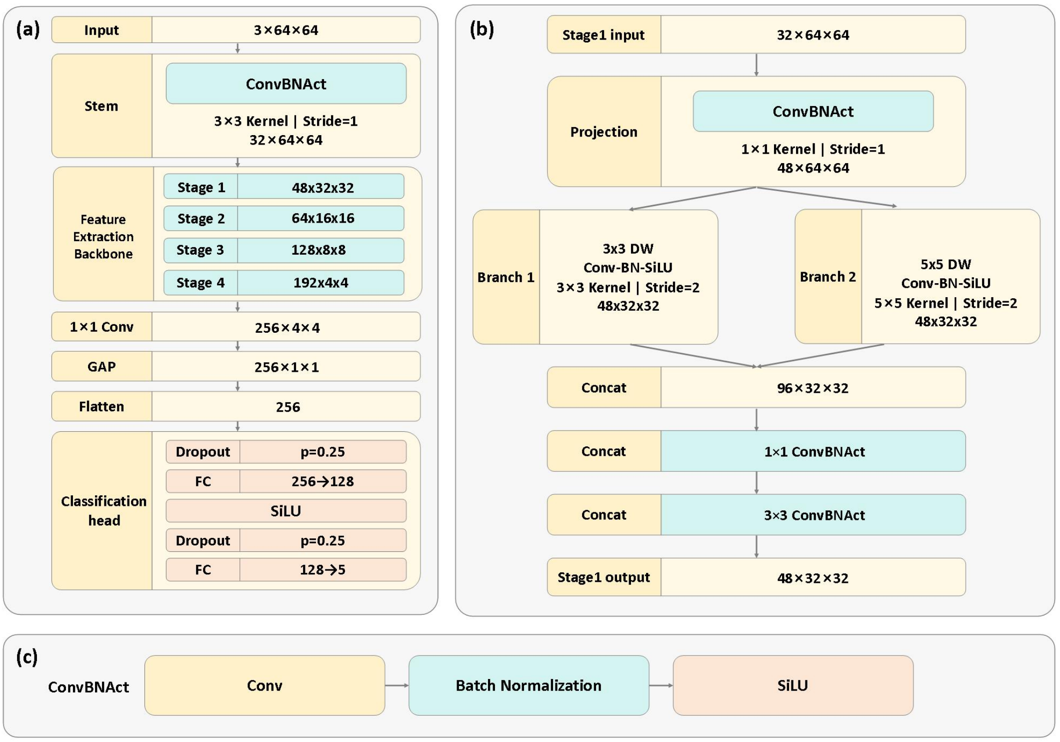

Figure S2. Detailed architecture of MSD-GasNet. (a) Layer-wise structure of the overall network, including the input tensor size, stem module, four-stage feature extraction backbone, 1×1 convolution, global average pooling, flattening operation, and classification head. (b) Internal structure of Stage 1, showing the projection layer, parallel 3×3 and 5×5 depthwise convolution branches, feature concatenation, 1×1 feature fusion, and 3×3 convolutional refinement. (c) Composition of the ConvBNAct unit, which consists of convolution, batch normalization, and SiLU activation.

Supporting Information S3 – Hardware and Software Setup

All experiments were performed on a local workstation equipped with an 11th Gen Intel(R) Core(TM) i7-1165G7 CPU. The proposed MSD-GasNet and all baseline models were implemented in Python 3.11.3 with PyTorch 2.11.0+cpu.

Data preprocessing, GASF image generation, model training, inference-time measurement, and performance evaluation were conducted under the same CPU-only environment. This setting ensured a fair comparison of computational efficiency among different models. It also shows that the proposed model does not rely on

high-performance GPU resources, supporting its potential applicability to resource-constrained VOC recognition and portable electronic-nose systems.

Supporting Information S4 – Training Settings and Baseline Models on Dataset 1

S4.1 Training Processes of Deep Learning Models on Dataset 1

All models were implemented in PyTorch and trained under the same experimental protocol to ensure a fair comparison. The input GASF images were resized to 64×64 pixels and normalized before being fed into the models. For the five-class VOC classification task, cross-entropy loss was used as the objective function, and Adam was adopted for parameter optimization.

To evaluate the effectiveness of MSD-GasNet, several representative deep learning models were included for comparison. ResNet50, VGG19, MobileNetV3-large, and AlexNet were selected as conventional CNN baselines with different architectural characteristics, including residual learning, deep standard convolutional stacking, lightweight mobile-oriented convolution, and classical CNN design. To strengthen the comparison with more recent lightweight architectures, GhostNetV3-1.0, StarNet-S2, and FastViT-T8 were further added as additional baseline models. For all baseline models, the final classification layer was modified to output five VOC categories. In addition, MSD-GasNet 1 was included as an ablation variant of the proposed model, in which the multi-scale branch design was replaced by a single-scale depthwise convolutional structure.

The training loss and accuracy curves of the baseline models and the ablation variant on Dataset 1 are provided in Figure S3 – S10. These supplementary curves were recorded under comparable training settings and are used to further compare the convergence behavior of different architectures on the GASF-based VOC classification task.

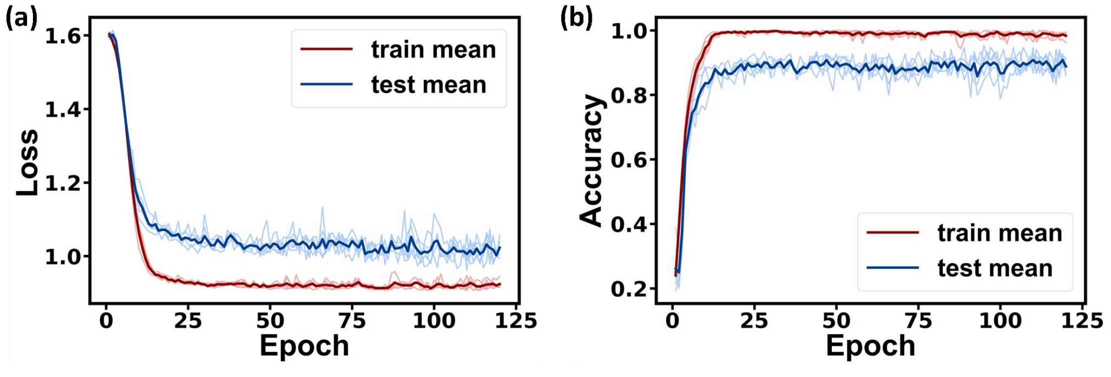

Figure S3. Training process of ResNet50 on Dataset 1: (a) loss curves and (b) accuracy curves.

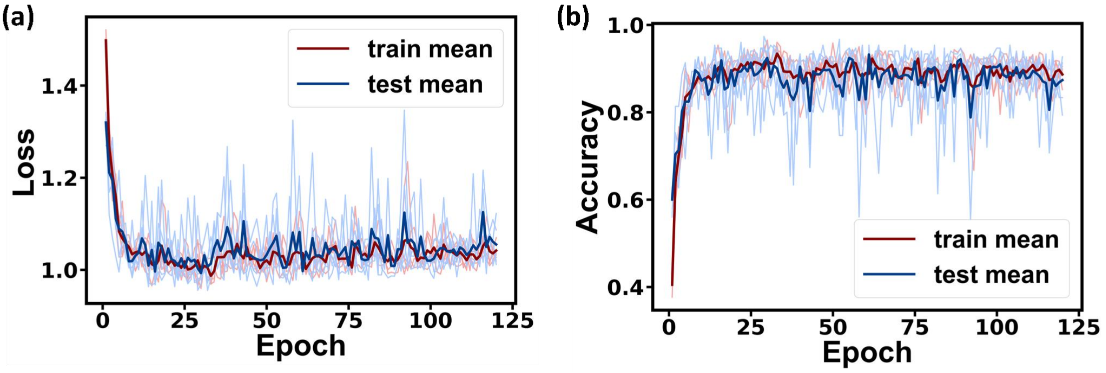

Figure S4. Training process of VGG19 on Dataset 1: (a) loss curves and (b) accuracy curves.

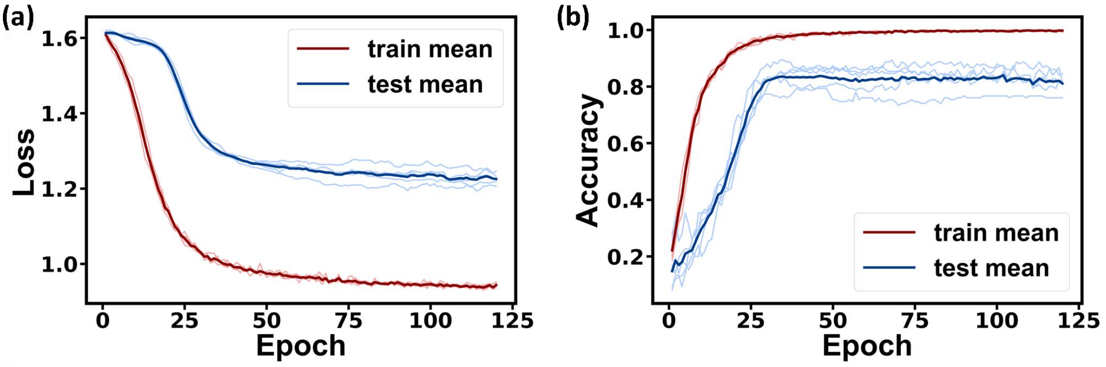

Figure S5. Training process of MobileNetV3-large on Dataset 1: (a) loss curves and (b) accuracy curves.

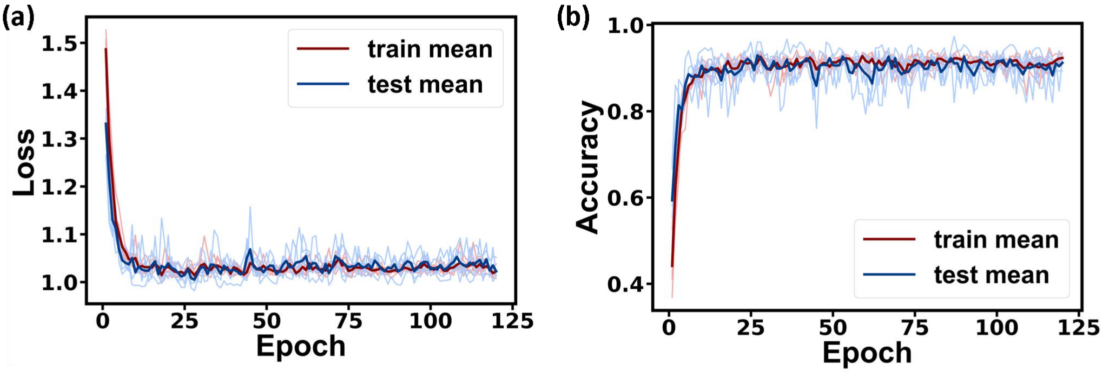

Figure S6. Training process of AlexNet on Dataset 1: (a) loss curves and (b) accuracy curves.

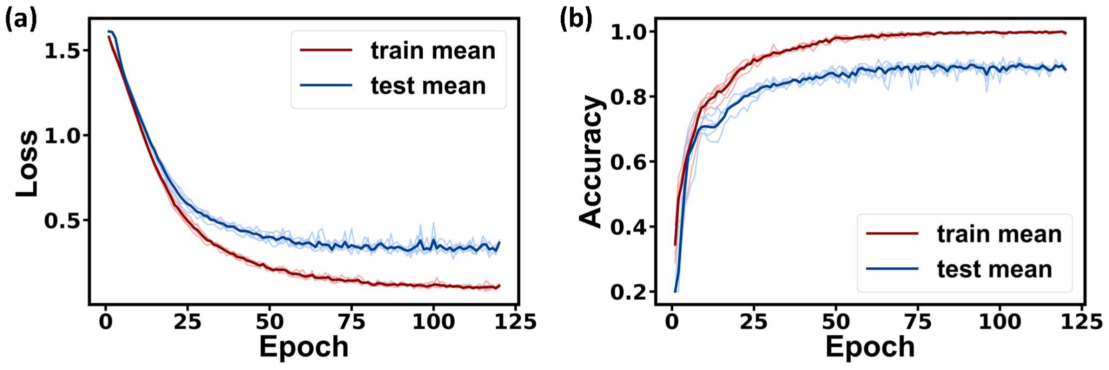

Figure S7. Training process of MSD-GasNet 1 on Dataset 1: (a) loss curves and (b) accuracy curves.

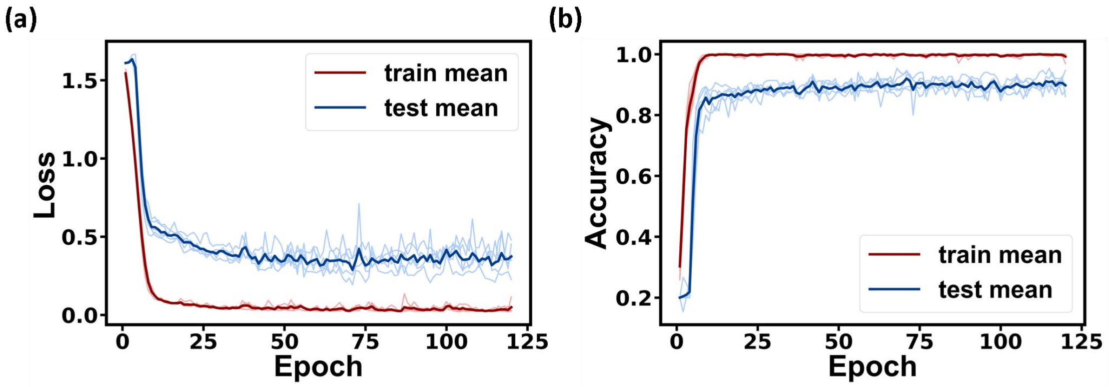

Figure S8. Training process of GhostNetV3-1.0 on Dataset 1: (a) loss curves and (b) accuracy curves.

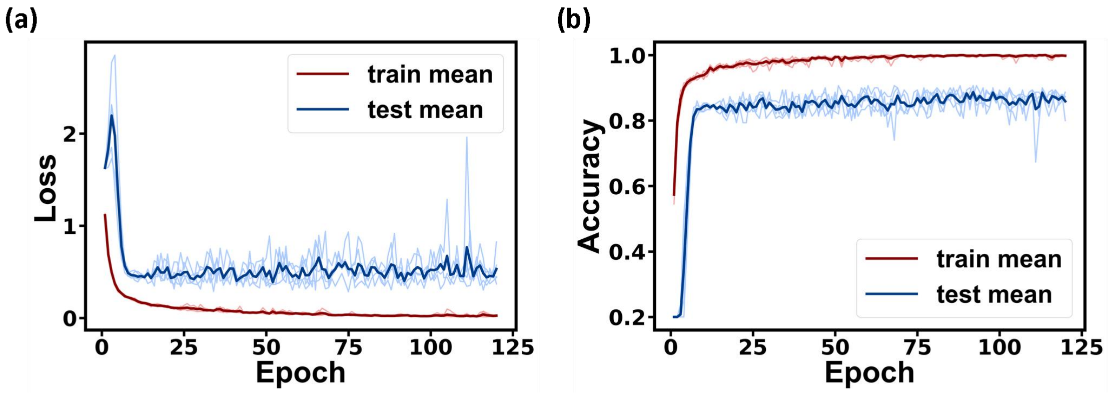

Figure S9. Training process of StarNet-S2 on Dataset 1: (a) loss curves and (b) accuracy curves.

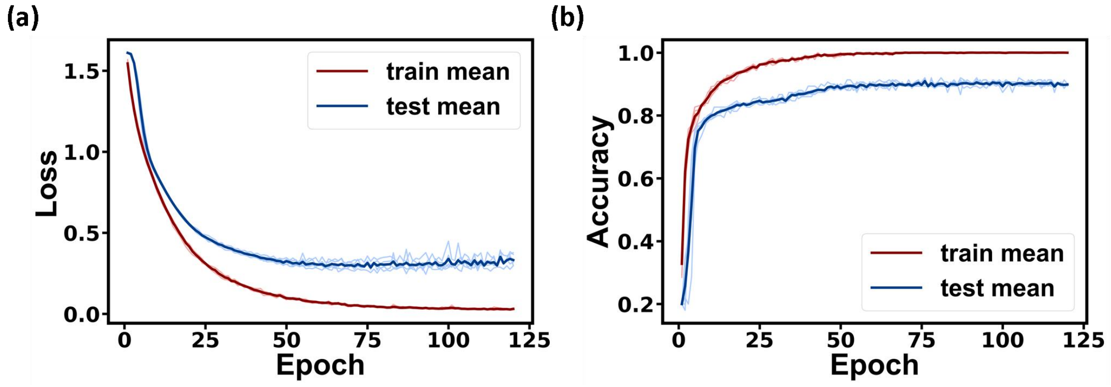

Figure S10. Training process of FastViT-T8 on Dataset 1: (a) loss curves and (b) accuracy curves.

S4.2 Classification Visualization on Dataset 1

To provide a more intuitive comparison of the classification behavior on Dataset 1, confusion matrices and t-SNE visualizations were further provided for different models. The confusion matrices of traditional machine learning classifiers, including Logistic Regression, Random Forest, Quadratic Discriminant Analysis, and k-Nearest Neighbors, are shown in Figure S11. These results provide additional references for evaluating the performance of conventional feature-based classifiers on the same five-class VOC classification task.

The confusion matrices of different deep learning models are shown in Figure S12, including MSD-GasNet, MSD-GasNet 1, ResNet50, VGG19, MobileNetV3-large, AlexNet, GhostNetV3-1.0, StarNet-S2, and FastViT-T8. Compared with the baseline models and the ablation variant, MSD-GasNet shows clearer diagonal dominance, indicating fewer misclassifications among the five VOC categories.

In addition, the t-SNE visualizations of the learned feature distributions are provided in Figure S13. These visualizations further illustrate the feature separability of different deep learning models on Dataset 1. The features extracted by MSD-GasNet show relatively compact intra-class distributions and clearer inter-class separation, supporting the effectiveness of the proposed multi-scale feature extraction design.

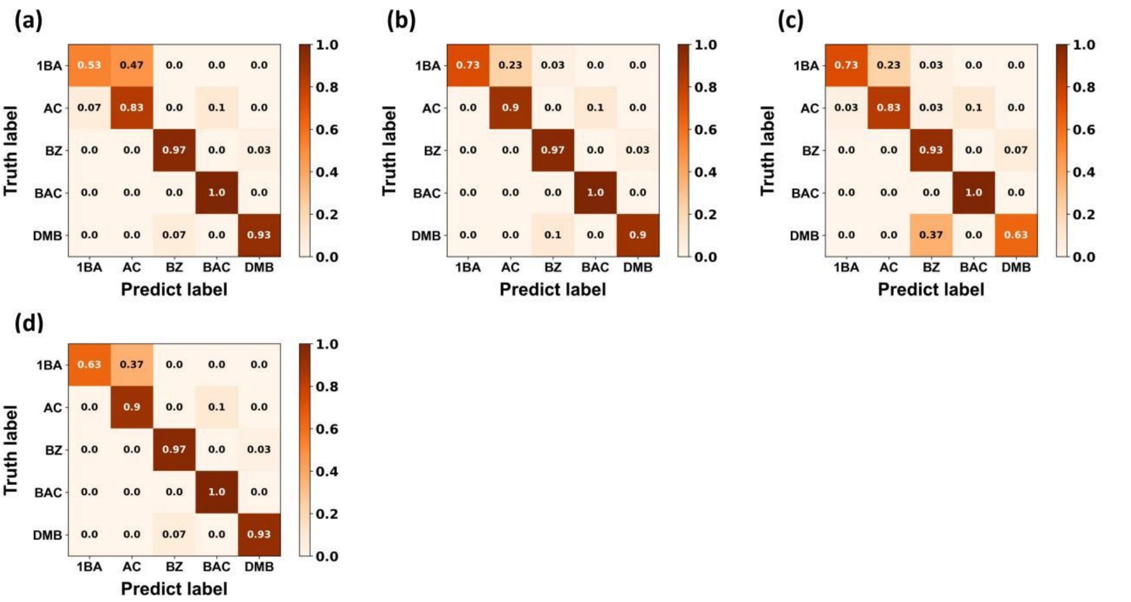

Figure S11. Confusion matrices of traditional machine learning classifiers on Dataset 1: (a) Logistic Regression, (b) Random Forest, (c) Quadratic Discriminant Analysis, and (d) k-Nearest Neighbors.

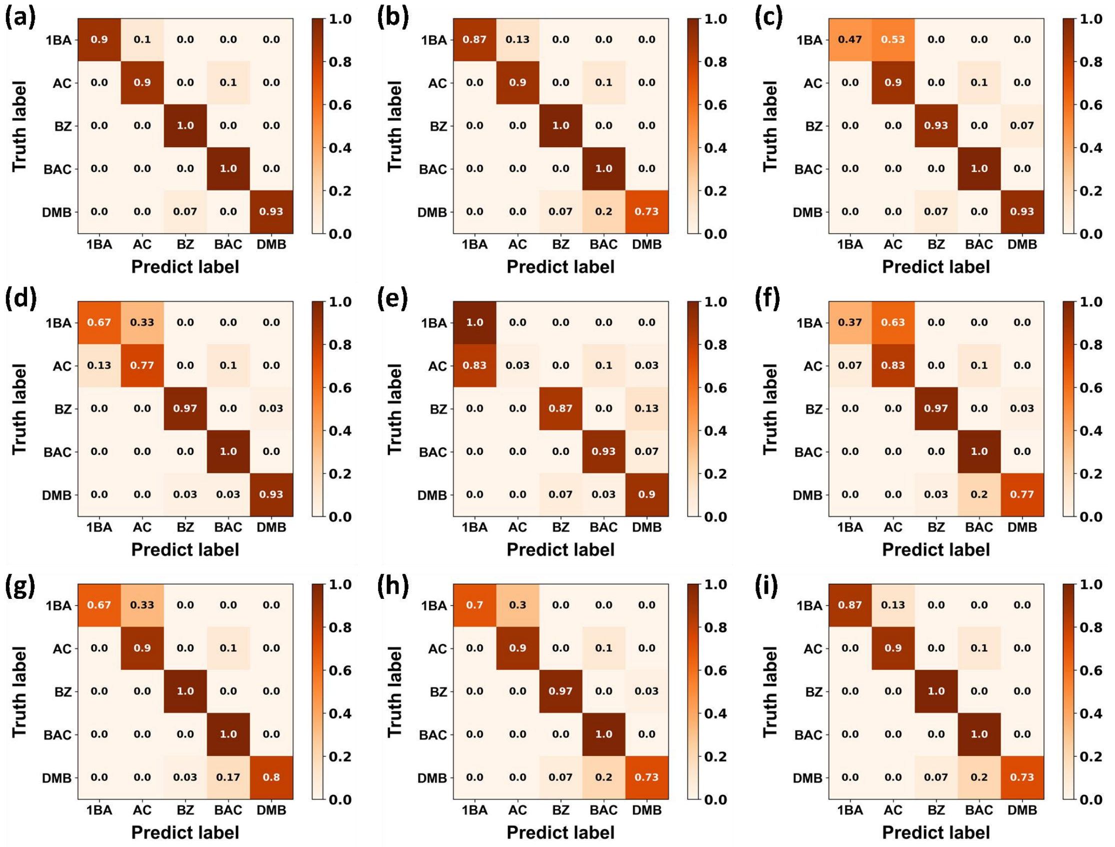

Figure S12. Confusion matrices of different deep learning models on Dataset 1: (a) MSD-GasNet, (b) MSD-GasNet 1, (c) ResNet50, (d) VGG19, (e) MobileNetV3-large, (f) AlexNet, (g) GhostNetV3-1.0, (h) StarNet-S2, and (i) FastViT-T8.

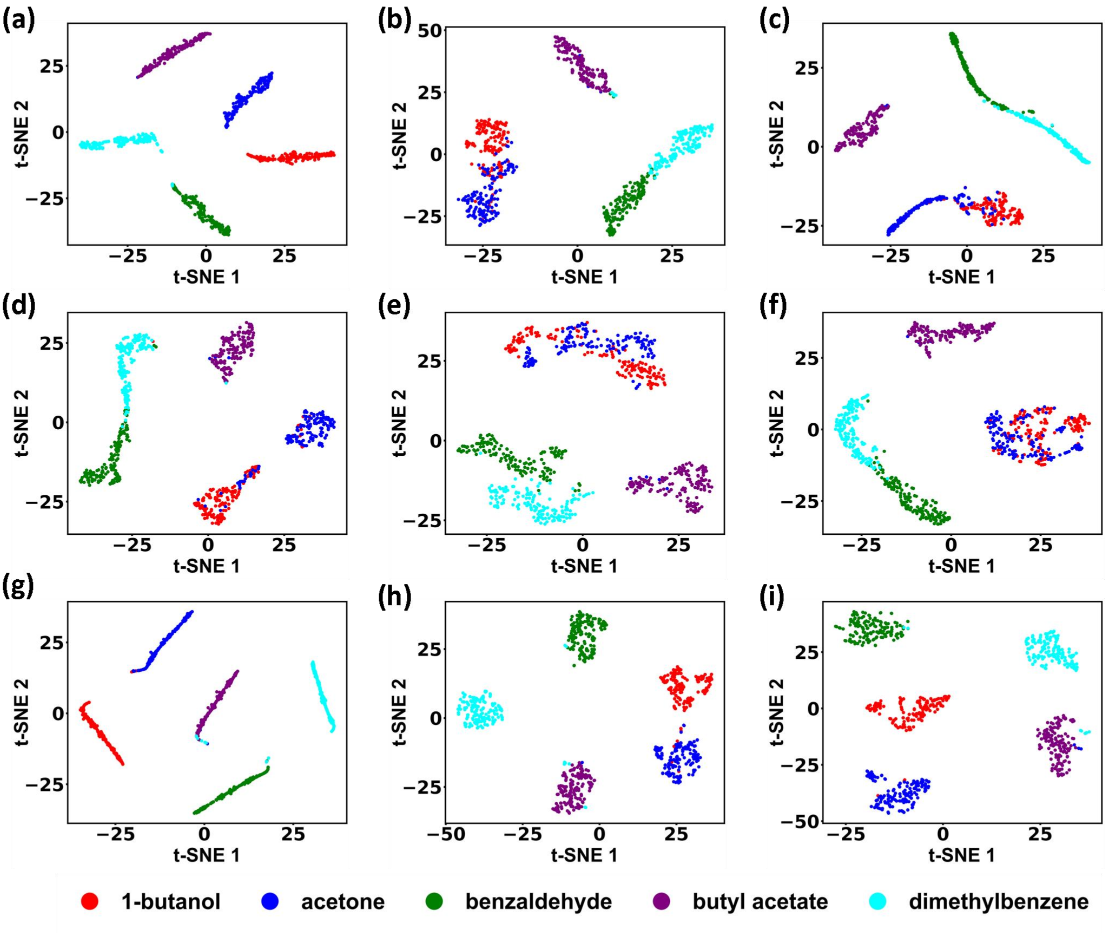

Figure S13. t-SNE visualization of learned feature distributions extracted by different deep learning models on Dataset 1: (a) MSD-GasNet, (b) MSD-GasNet 1, (c) ResNet50, (d) VGG19, (e) MobileNetV3-large, (f) AlexNet, (g) GhostNetV3-1.0, (h) StarNet-S2, and (i) FastViT-T8.

Supporting Information S5–Independent Validation on Dataset 2

S5.1 Hyperparameter Settings and Quantitative Results on Dataset 2

To further evaluate the generalization ability of the proposed method under more independent experimental conditions, Dataset 2 was used as an additional validation dataset. In this experiment, five representative VOCs were selected from Dataset 2, namely acetone, benzaldehyde, butyl acetate, dimethylbenzene, and methanol. The first five complete experimental rounds were used as the training set, while the last five complete experimental rounds were used as the testing set. This chronological splitting strategy avoids randomly mixing samples from the same experimental round into both the training and testing sets, thereby providing a more rigorous evaluation of model generalization.

The hyperparameter configurations of different deep learning models used in the independent validation experiment on Dataset 2 are summarized in Table S2. The classification performance comparison among traditional machine learning methods, CNN baselines, lightweight networks, the ablation model, and MSD-GasNet is presented in Table S3. In addition, the computational efficiency of different deep learning models, including parameter number, training time, and single-sample inference time, is summarized in Table S4. These supplementary results provide quantitative evidence for evaluating the accuracy, stability, model compactness, and inference efficiency of MSD-GasNet on the independent validation dataset.

Table S2. Hyperparameter configurations of different deep learning models in the independent validation experiment on Dataset 2.

Model/Hyperparameter	epoch	batch size	initial learning rate	optimizer	betas	weight_decay	step_size	image size
ResNet50	150	48	0.00008	Adam	(0.9,0.999)	0.1	20	64×64
VGG19	150	48	0.00004	Adam	(0.9,0.999)	0.1	20	64×64
MobilenetV3-large	2000	48	0.00008	Adam	(0.9,0.999)	0.1	20	64×64
AlexNet	150	48	0.00006	Adam	(0.9,0.999)	0.1	20	64×64
GhostNetV3-1.0	180	48	0.00008	Adam	(0.9,0.999)	0.1	20	64×64
StarNet-S2	180	48	0.00004	Adam	(0.9,0.999)	0.1	20	64×64
FastViT-T8	180	48	0.00004	Adam	(0.9,0.999)	0.1	20	64×64
MSD-GasNet 1	150	48	0.00015	Adam	(0.9,0.999)	0.1	20	64×64
MSD-GasNet	150	48	0.00002	Adam	(0.9,0.999)	0.1	20	64×64

Table S3. Classification performance comparison of traditional machine learning methods, CNN baselines, lightweight networks, the ablation model, and MSD-GasNet on Dataset 2.

Model	Accuracy Mean (%)	Accuracy Std. (%)
LR	77.28	2.06
RF	86.14	1.16
QDA	79.91	2.90
KNN	78.95	1.46
ResNet50	88.80	3.68
VGG19	91.36	5.69
MobilenetV3-large	93.78	1.34
AlexNet	93.93	0.77
GhostNetV3-1.0	77.34	3.92

StarNet-S2	87.79	3.85
FastViT-T8	89.45	1.47
MSD-GasNet 1	92.33	3.42
MSD-GasNet	95.12	1.11

Table S4. Computational efficiency comparison of different deep learning models on Dataset 2.

Model	Params.	Training Time (s)	Single Inference Time (ms)
ResNet50	23.5183 M	97.731288	8.775
VGG19	139.5907 M	174.753410	3.389
MobilenetV3-large	4.2084 M	964.404748	5.904
AlexNet	57.0243 M	75.920441	2.605
GhostNetV3-1.0	6.8544 M	204.928281	3.369
StarNet-S2	3.4263 M	78.724953	2.650
FastViT-T8	3.2611 M	90.439588	3.412
MSD-GasNet	796.6450 K	66.978243	2.170

S5.2 Training Processes of Deep Learning Models on Dataset 2

The training loss and accuracy curves of different deep learning models on Dataset 2 are provided in Figures S14–S22. These models include MSD-GasNet, MSD-GasNet 1, ResNet50, VGG19, MobileNetV3-large, AlexNet, GhostNetV3-1.0, StarNet-S2, and FastViT-T8. The curves were used to examine whether the compared models could be trained stably under the independent validation setting of Dataset 2.

As shown in these figures, the compared models generally show decreasing training loss and increasing classification accuracy during training. Among them, MSD-GasNet achieves stable convergence under the chronological train/test split of Dataset 2, indicating that the proposed lightweight multi-scale structure can still be effectively optimized when the training and testing samples are separated by experimental rounds.

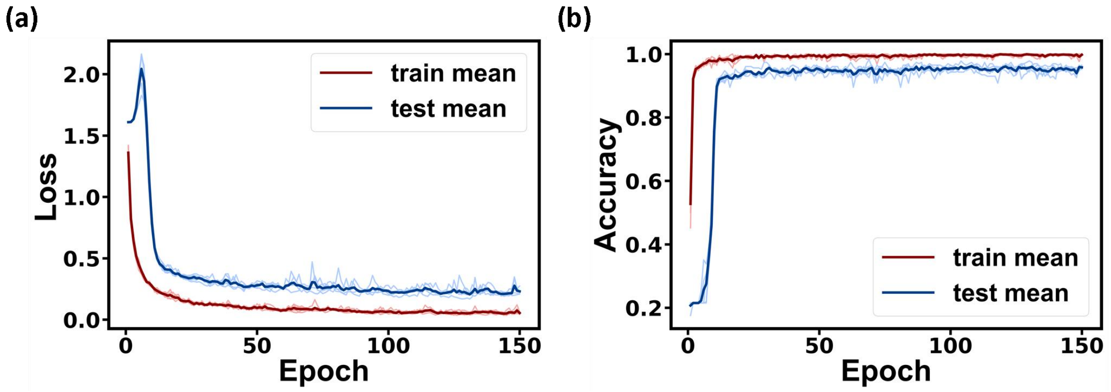

Figure S14. Training and testing process of MSD-GasNet on Dataset 2: (a) loss curves and (b) accuracy curves.

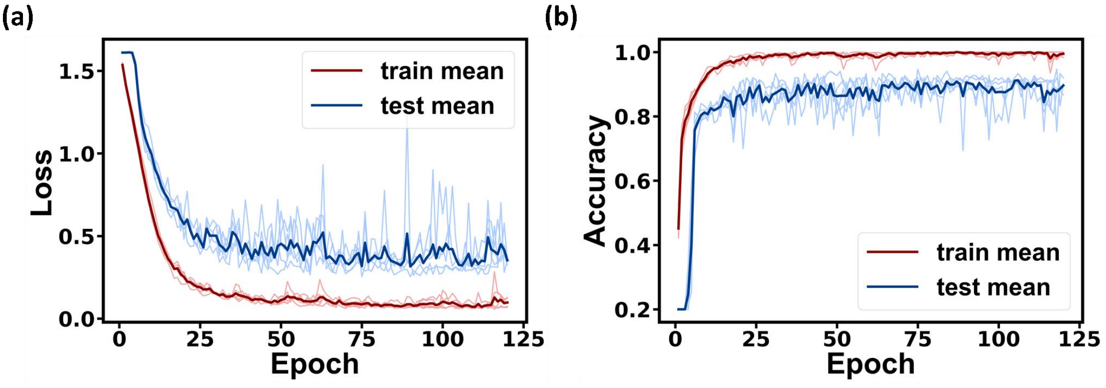

Figure S15. Training and testing process of MSD-GasNet 1 on Dataset 2: (a) loss curves and (b) accuracy curves.

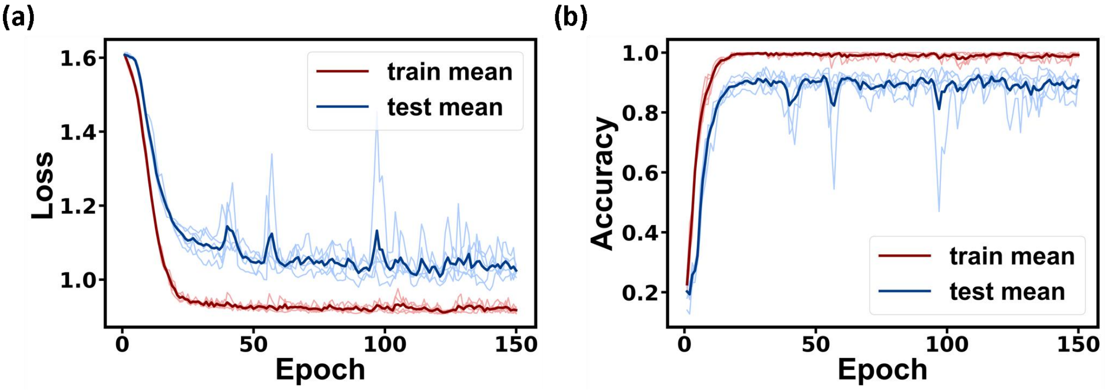

Figure S16. Training and testing process of ResNet50 on Dataset 2: (a) loss curves and (b) accuracy curves.

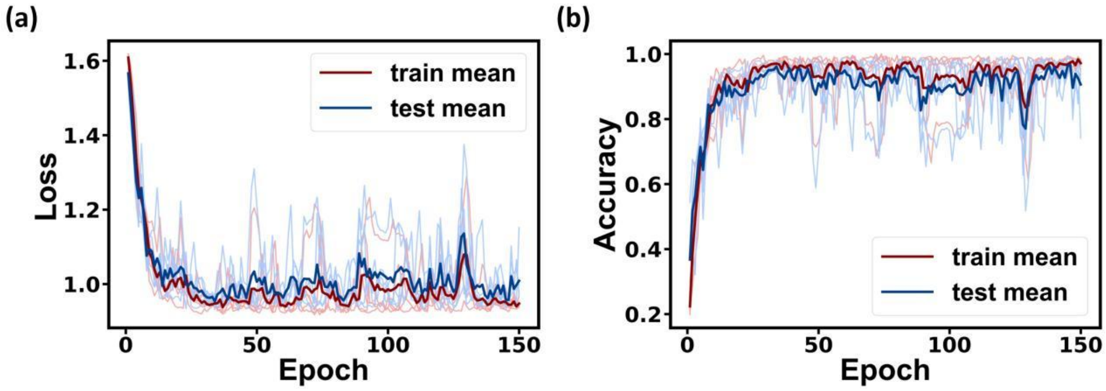

Figure S17. Training and testing process of VGG19 on Dataset 2: (a) loss curves and (b) accuracy curves.

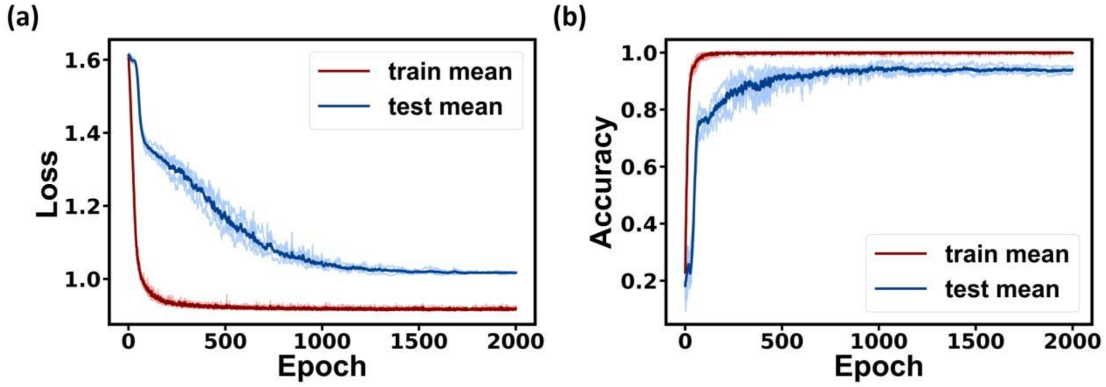

Figure S18. Training and testing process of MobileNetV3-large on Dataset 2: (a) loss curves and (b) accuracy curves.

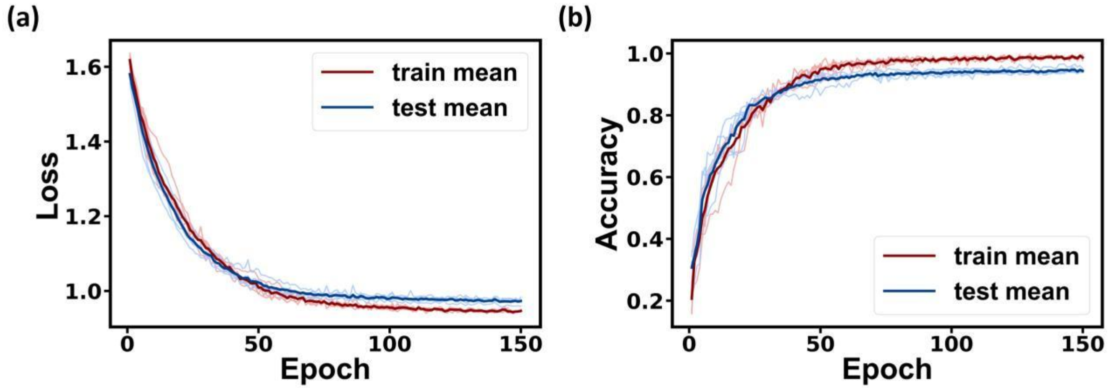

Figure S19. Training and testing process of AlexNet on Dataset 2: (a) loss curves and (b) accuracy curves.

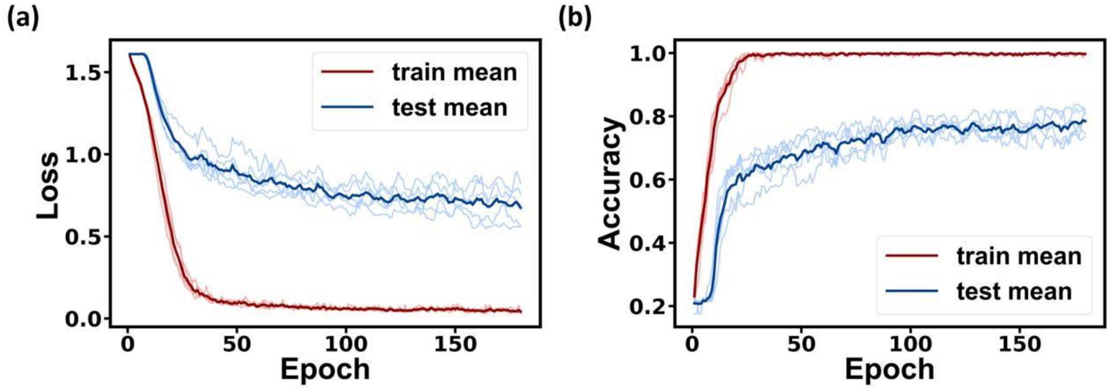

Figure S20. Training process of GhostNetV3-1.0 on Dataset 2: (a) loss curves and (b) accuracy curves.

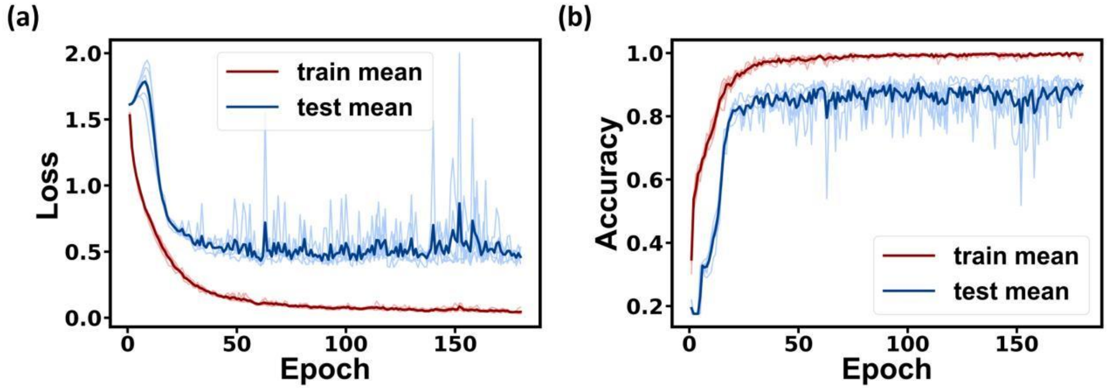

Figure S21. Training process of StarNet-S2 on Dataset 2: (a) loss curves and (b) accuracy curves.

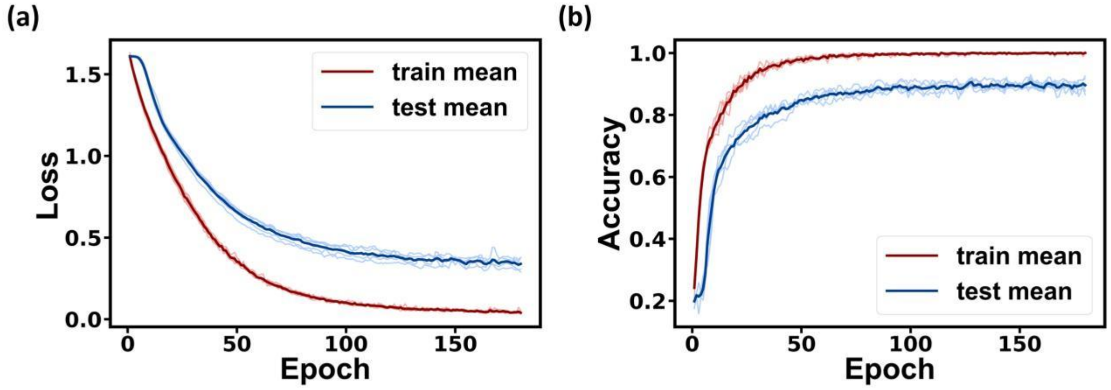

Figure S22. Training process of FastViT-T8 on Dataset 2: (a) loss curves and (b) accuracy curves.

S5.3 Classification Visualization on Dataset 2

To further analyze the classification behavior of different models on Dataset 2, confusion matrices and t-SNE visualizations were provided as supplementary evidence. The confusion matrices of traditional machine learning classifiers, including

Logistic Regression, Random Forest, Quadratic Discriminant Analysis, and k-Nearest Neighbors, are shown in Figure S23. These results provide a visual comparison of the classification performance of conventional machine learning methods under the independent validation setting.

The confusion matrices of different deep learning models on Dataset 2 are shown in Figure S24, including MSD-GasNet, MSD-GasNet 1, ResNet50, VGG19, MobileNetV3-large, AlexNet, GhostNetV3-1.0, StarNet-S2, and FastViT-T8. Compared with the baseline models and the ablation variant, MSD-GasNet shows clearer diagonal dominance, indicating stronger classification ability for the five selected VOC categories in Dataset 2.

In addition, the t-SNE visualizations of the learned feature distributions are shown in Figure S25. These visualizations provide an intuitive comparison of the feature separability achieved by different deep learning models. The features extracted by MSD-GasNet show relatively compact intra-class distributions and clearer inter-class separation, further supporting the effectiveness of the proposed lightweight multi-scale feature extraction design under the independent validation setting.

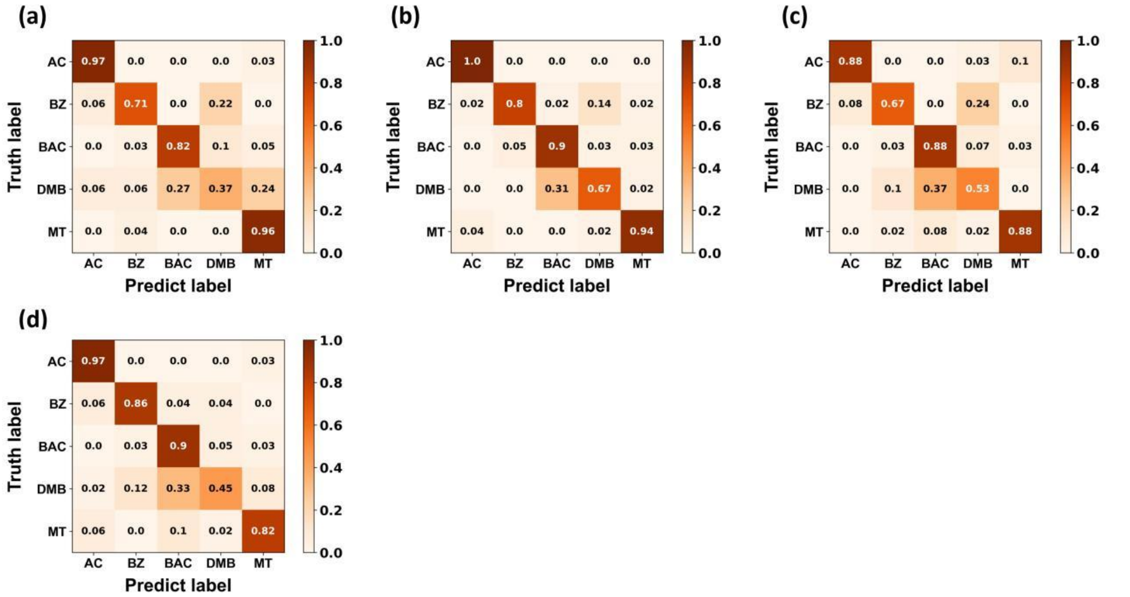

Figure S23. Confusion matrices of traditional machine learning classifiers on Dataset 2: (a) Logistic Regression, (b) Random Forest, (c) Quadratic Discriminant Analysis, and (d) k-Nearest Neighbors.

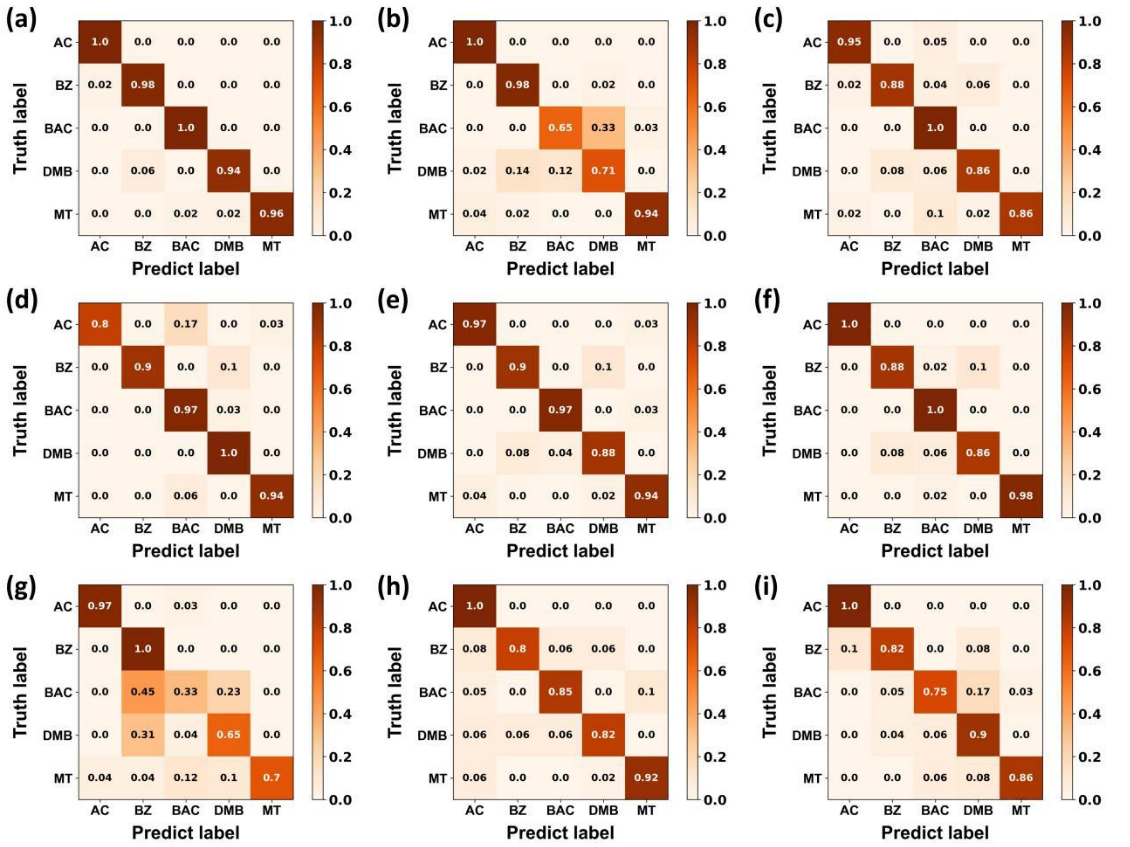

Figure S24. Confusion matrices of different deep learning models on Dataset 2: (a) MSD-GasNet, (b) MSD-GasNet 1, (c) ResNet50, (d) VGG19, (e) MobileNetV3-large, (f) AlexNet, (g) GhostNetV3-1.0, (h) StarNet-S2, and (i) FastViT-T8.

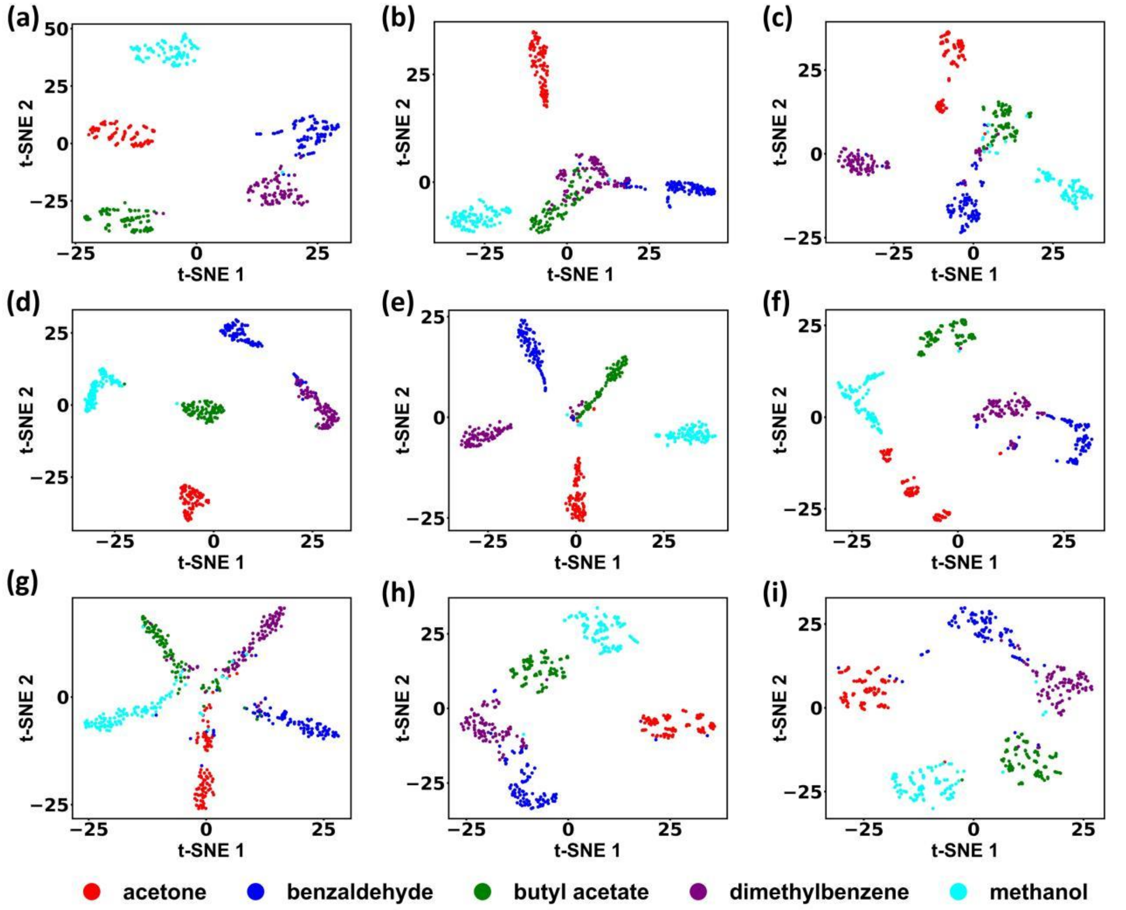

Figure S25. t-SNE visualization of learned feature distributions extracted by different deep learning models on Dataset 2: (a) MSD-GasNet, (b) MSD-GasNet 1, (c) ResNet50, (d) VGG19, (e) MobileNetV3-large, (f) AlexNet, (g) GhostNetV3-1.0, (h) StarNet-S2, and (i) FastViT-T8.

Supporting Information S6 – Comparison of Time-Series-to-Image Encoding Methods

To further justify the use of GASF encoding in this work, additional experiments were conducted by comparing different time-series-to-image encoding methods, including continuous wavelet transform (CWT), Markov transition field (MTF), short-time Fourier transform (STFT), and GASF. Representative images generated by CWT, MTF, and STFT are shown in Figure S26. These images illustrate that different encoding methods transform the same gas-sensing response sequence into different two-dimensional representations.

The proposed MSD-GasNet was then trained and evaluated using the images

generated by different encoding methods under the same five-class VOC classification setting on Dataset 1. The training loss and accuracy curves of MSD-GasNet using CWT, MTF, and STFT images are shown in Figures S27–S29, respectively. The quantitative results of different encoding methods are summarized in Table S5. Compared with CWT, MTF, and STFT, GASF achieved the highest mean accuracy and the lowest accuracy standard deviation. Although STFT showed slightly shorter training and inference times, GASF provided substantially better and more stable classification performance while maintaining a comparable computational cost. Therefore, GASF was selected as the main time-series-to-image encoding method in this study.

Table S5. Performance comparison of different time-series-to-image encoding methods using the proposed MSD-GasNet on Dataset 1.

Image type	Accuracy Mean (%)	Accuracy Std. (%)	Training Time (s)	Single Inference Time (ms)
CWT	86.18	5.10	95.932992	3.138
MTF	79.78	0.95	94.384965	3.092
STFT	88.75	4.83	89.040959	2.393
GASF	96.80	0.78	94.001315	2.537

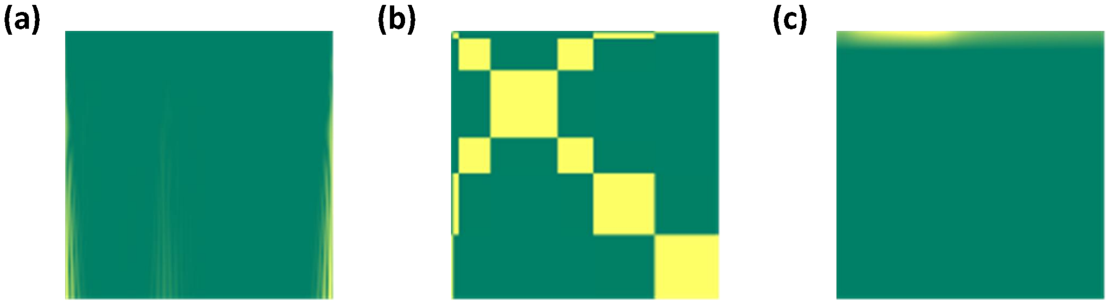

Figure S26. Representative images generated by different time-series-to-image encoding methods: (a) CWT, (b) MTF, and (c) STFT.

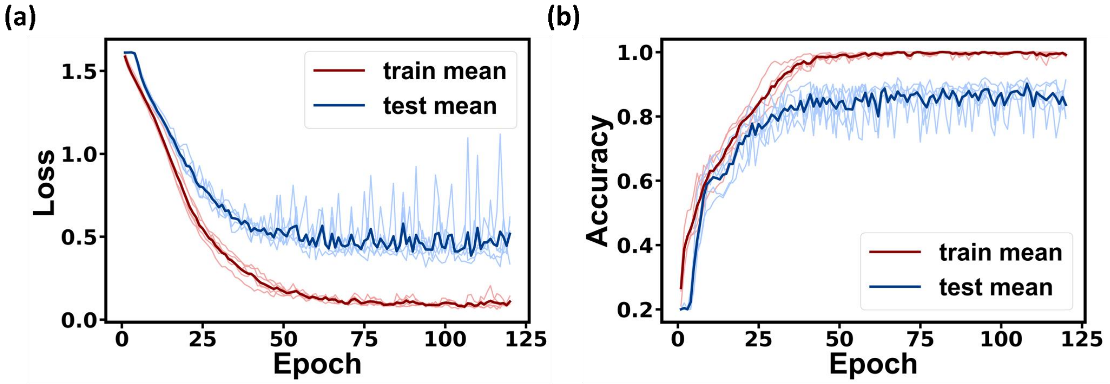

Figure S27. Training process of MSD-GasNet using CWT images on Dataset 1: (a)

loss curves and (b) accuracy curves.

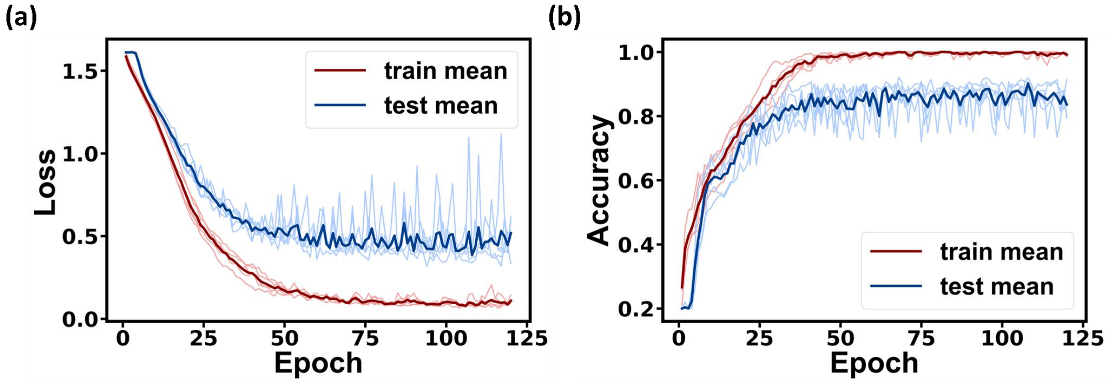

Figure S28. Training process of MSD-GasNet using MTF images on Dataset 1: (a) loss curves and (b) accuracy curves.

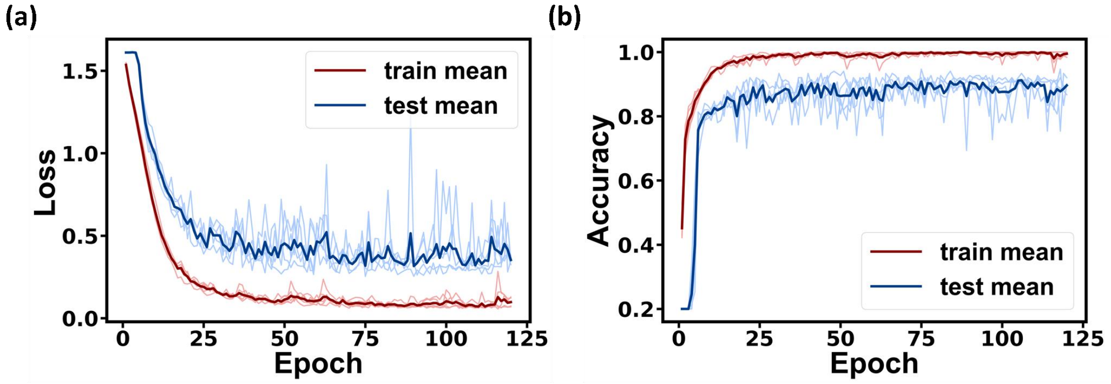

Figure S29. Training process of MSD-GasNet using STFT images on Dataset 1: (a) loss curves and (b) accuracy curves.

Supporting Information S7–Scalability Evaluation on All 12 VOC Categories in Dataset 1

To further evaluate whether the proposed method can be extended beyond the selected five representative VOCs, an additional scalability experiment was conducted using all 12 VOC categories in Dataset 1. Compared with the five-class setting, the 12-class task is more challenging because the number of target categories increases and some VOCs may have more similar response characteristics, which makes the classification boundaries more complex.

In this expanded 12-class VOC classification task, MSD-GasNet achieved a test accuracy of 87.01 ± 2.40%, with an average training time of 195.061 s and a single-sample inference time of 2.494 ms. Although the accuracy decreased compared with the five-class setting, the model still maintained reasonable classification performance under a substantially expanded category set. This result indicates that the

proposed GASF-based lightweight multi-scale framework is not limited to the selected five VOCs and has preliminary scalability to a larger VOC classification task.

The training loss and accuracy curves of MSD-GasNet for the 12-class VOC classification task are shown in Figure S30. The confusion matrix is shown in Figure S31, and the t-SNE visualization of the learned feature distributions is shown in Figure S32. These supplementary visualizations further illustrate the training behavior, class-level prediction results, and feature distribution characteristics of MSD-GasNet under the expanded 12-class setting.

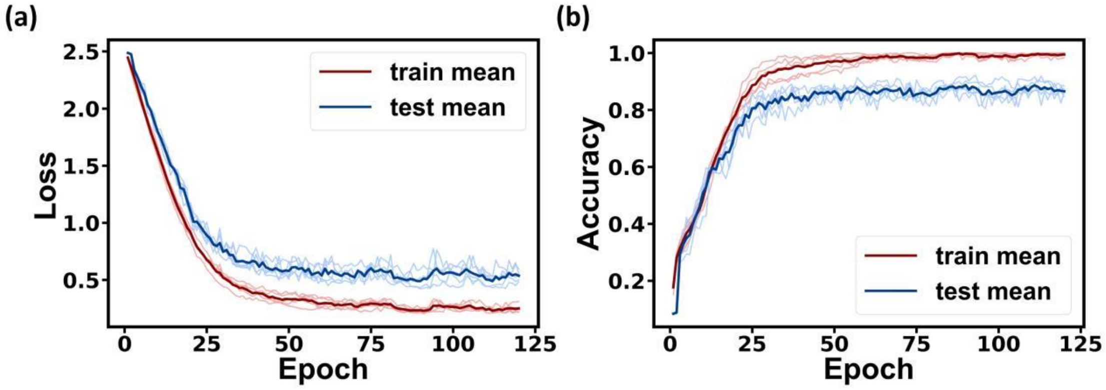

Figure S30. Training process of MSD-GasNet for the 12-class VOC classification task on Dataset 1: (a) loss curves and (b) accuracy curves.

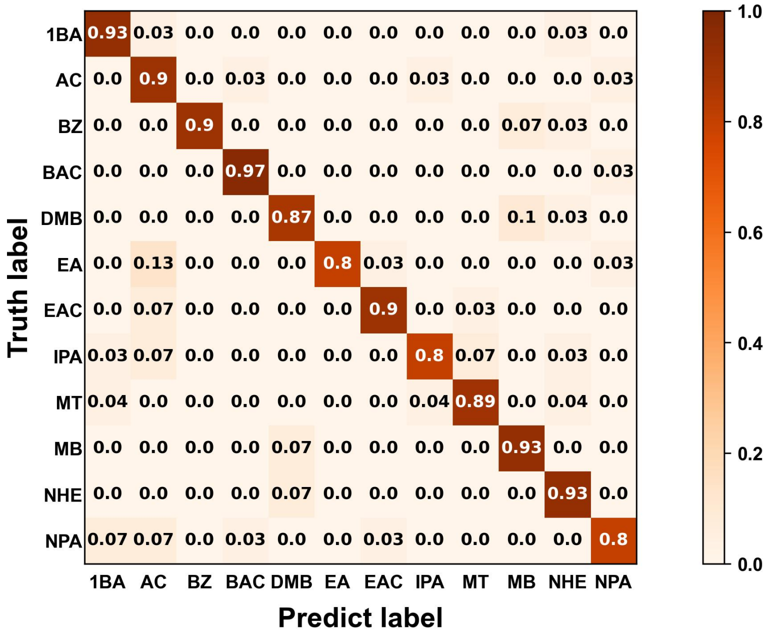

Figure S31. Confusion matrix of MSD-GasNet for the 12-class VOC classification task on Dataset 1.

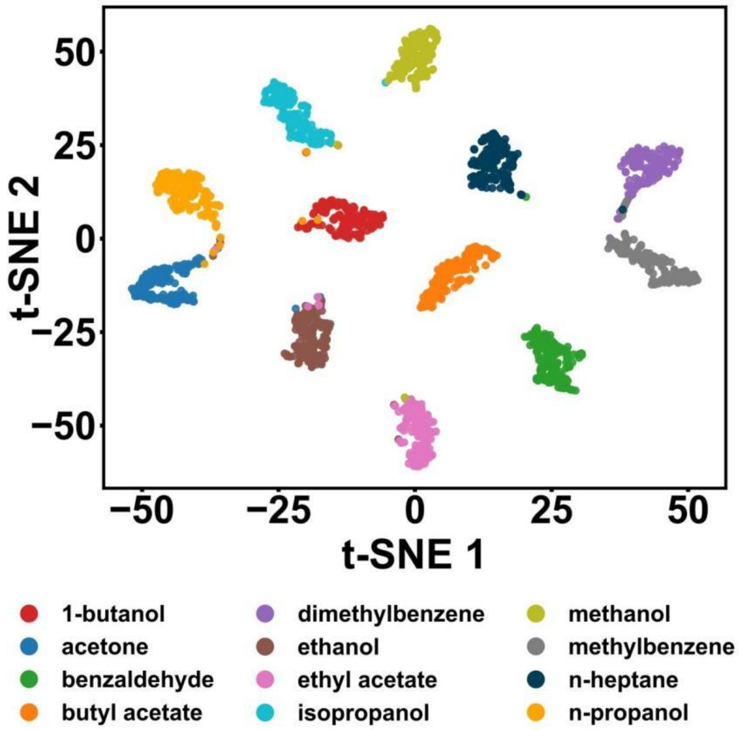

Figure S32. t-SNE visualization of learned feature distributions extracted by MSD-GasNet for the 12-class VOC classification task on Dataset 1.
